# Genetic and Ecological Divergence Between Northwest Atlantic Killer Whale Populations

**DOI:** 10.1002/ece3.73593

**Published:** 2026-04-30

**Authors:** Caila E. Kucheravy, Evelien de Greef, Steven H. Ferguson, Cortney A. Watt, Jack W. Lawson, Jeremy J. Kiszka, Colin J. Garroway, Cory J. D. Matthews

**Affiliations:** ^1^ Department of Biological Sciences University of Manitoba Winnipeg Manitoba Canada; ^2^ Arctic Fisheries and Marine Mammal Science Division Fisheries and Oceans Canada Winnipeg Manitoba Canada; ^3^ Northwest Atlantic Fisheries Centre Fisheries and Oceans Canada St. John's Newfoundland and Labrador Canada; ^4^ Institute of Environment, Department of Biological Sciences Florida International University Miami Florida USA

**Keywords:** compound‐specific stable isotope analysis, diet, distribution, *Orcinus orca*, population structure, whole genome sequencing

## Abstract

Killer whales ( *Orcinus orca* ) exhibit substantial genetic and ecological variation across their global distribution. Differentiation between neighboring or sympatric populations is thought to be driven by foraging specialization and social organization, which can lead to reproductive isolation and facilitate the emergence of distinct ecotypes or morphotypes. Here, we use whole‐genome resequencing and compound‐specific stable isotope analysis of amino acids to investigate links between genetic and ecological differentiation in two genetically distinct killer whale populations in the northwest Atlantic, specifically in the eastern Canadian Arctic and Greenland (ECAG1 and ECAG2). Essential amino acid stable carbon isotope ratios (δ^13^C) suggest that the populations maintain largely distinct distributions or habitat use patterns. Amino acid‐specific stable nitrogen isotope ratios (δ^15^N) indicate ECAG1 has a higher trophic level diet than ECAG2. Previously undetected genetic substructure within the ECAG1 population revealed finer‐scale genetic differentiation between individuals sampled in the eastern Canadian Arctic and those sampled in more temperate northwest Atlantic waters. However, small sample sizes prevented exploration of isotopic differentiation among them. Within ECAG1, considerable interannual variation in δ^13^C and δ^15^N amino acid values of seven individuals sampled across different years suggests some degree of ecological plasticity. Concurrent genetic and ecological differentiation suggests that northwest Atlantic killer whales have diverged ecologically, possibly in allopatry, and are now reproductively isolated under secondary contact, comparable to population‐level differences observed in other regions. However, their degree of ecological plasticity and secondary contact within expanding Arctic ranges raises questions about whether current levels of divergence will be maintained or eroded with ongoing Arctic warming.

## Graphical abstract

Killer whale (*Orcinus orca*) populations exhibit substantial genetic, ecological, and morphological differences across their global distribution. In this study, we use whole‐genome resequencing and compound‐specific stable isotope analysis of amino acids to show concurrent genetic and ecological differentiation in two relatively understudied killer whale populations in the northwest Atlantic, and discuss these results in the context of ongoing Arctic warming.

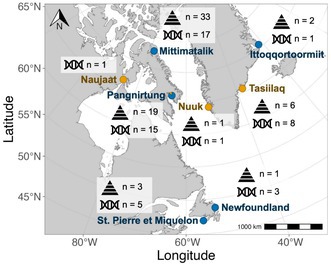

## 1 Introduction

Species with widespread distributions often become locally adapted to different environmental conditions (Futuyma and Moreno 1988). Ecological specialization and behavioral divergence among groups can eventually lead to reproductive isolation, which in turn can lead to phenotypic and genetic differentiation (Dieckmann and Doebeli 1999; McKinnon et al. 2004). Populations that have diverged in allopatry can come into secondary contact following range shifts. If differentiation in allopatry has progressed to the point where populations no longer recognize each other as mates, or interbreeding between populations produces less fit offspring, population divergence is expected to continue in sympatry (Servedio and Noor 2003; Grant and Grant 2009). Such highly differentiated populations within species are often referred to as “ecotypes,” a term used to describe an advanced stage of evolutionary divergence, whereby a population becomes genetically, ecologically, and behaviorally distinct due to adaptations to a given environment (Turesson 1922; de Bruyn et al. 2013). Identifying emerging ecotypes is important for understanding the speciation process and informing the conservation and management of differently adapted populations (Stronen et al. 2022).

Killer whales ( *Orcinus orca* ) exhibit significant ecological, morphological, and genetic differences across their global distribution (Ford 2009; Foote et al. 2009; Morin et al. 2010). Killer whale ecotypes were first identified in the eastern North Pacific, where neighboring and sympatric ecotypes differ in diet and foraging behavior, morphology and coloration, social structure and acoustic behavior, and movement patterns, with limited or no gene flow despite their close physical proximity (Ford 2009; de Bruyn et al. 2013). Two of these ecotypes, Bigg's killer whales and Resident killer whales, have been proposed to be distinct species (Morin et al. 2024) but are formally recognized as subspecies (Society for Marine Mammalogy 2024). Other killer whale populations in this region and elsewhere also exhibit varying degrees of differentiation across these characteristics (e.g., Pitman and Ensor 2003; Tavares et al. 2018; Jourdain et al. 2020, 2024; Van Cise et al. 2024). Ecological niche divergence is considered the primary driver of ecotypic divergence in killer whales, with divergence perpetuated by social organization and learning within groups (Hoelzel et al. 2007; Foote et al. 2009, 2016). There remains, however, considerable debate about the degree of divergence that merits classification of ecologically and genetically diverged populations as separate killer whale ecotypes, subspecies, or species (Morin et al. 2010, 2024; Riesch et al. 2012).

In the eastern Canadian Arctic, where killer whales are seasonally resident during the ice‐free summer months (Higdon et al. 2012), genomic analyses have identified two populations that are as genetically distinct as killer whale ecotypes found elsewhere (Garroway et al. 2024). These populations have been called “Eastern Canadian Arctic and Greenland 1” (ECAG1), comprising individuals sampled in the Canadian High Arctic and Newfoundland, and “Eastern Canadian Arctic and Greenland 2” (ECAG2), comprising individuals sampled in the Canadian Low Arctic and East Greenland (Garroway et al. 2024). Ecological differentiation among killer whales sampled throughout the Canadian Arctic and Newfoundland has also been inferred from measurements of stable isotopes and other dietary proxies (e.g., contaminant loads), which indicate differences in distribution and diet (Matthews and Ferguson 2014; Matthews et al. 2024; Matthews, Lawson, and Ferguson 2021; Matthews, Longstaffe, et al. 2021; Desforges et al. 2024) comparable to population‐level variation observed in other regions (Krahn et al. 2007; Lawson et al. 2020; Matthews, Lawson, and Ferguson 2021).

In the northwest Atlantic, it is critical to advance our understanding of the ecological roles of these populations, which are increasingly present in the Arctic (de Bruyn et al. 2013; Donelson et al. 2019; Monaco et al. 2020). Here, we expand genomic (Garroway et al. 2024) and compound‐specific stable isotope (Matthews, Lawson, and Ferguson 2021; Matthews et al. 2024) datasets with additional samples to directly investigate ecological differentiation between genetic populations of killer whales in the northwest Atlantic. First, we assessed genetic population structure using whole genome sequences. Second, we examined ecological differentiation between genetic populations, inferring distribution and/or habitat differences using stable carbon isotope ratios (δ^13^C) of essential amino acids (AA_ESS_) (Hare et al. 1991; Larsen et al. 2013; McMahon et al. 2016), and trophic differences using amino acid (AA)‐specific stable nitrogen isotope ratios (δ^15^N) (Matthews, Lawson, and Ferguson 2021; Matthews et al. 2024). Finally, we explored within‐population variation by comparing δ^13^C AA_ESS_ and δ^15^N AA values of individuals that were sampled across multiple years. We reveal ecological differentiation between genetic populations and discuss potential ecological and evolutionary implications in the context of their Arctic expansion.

## 2 Methods

### 2.1 Sample Collection

**FIGURE 1:**
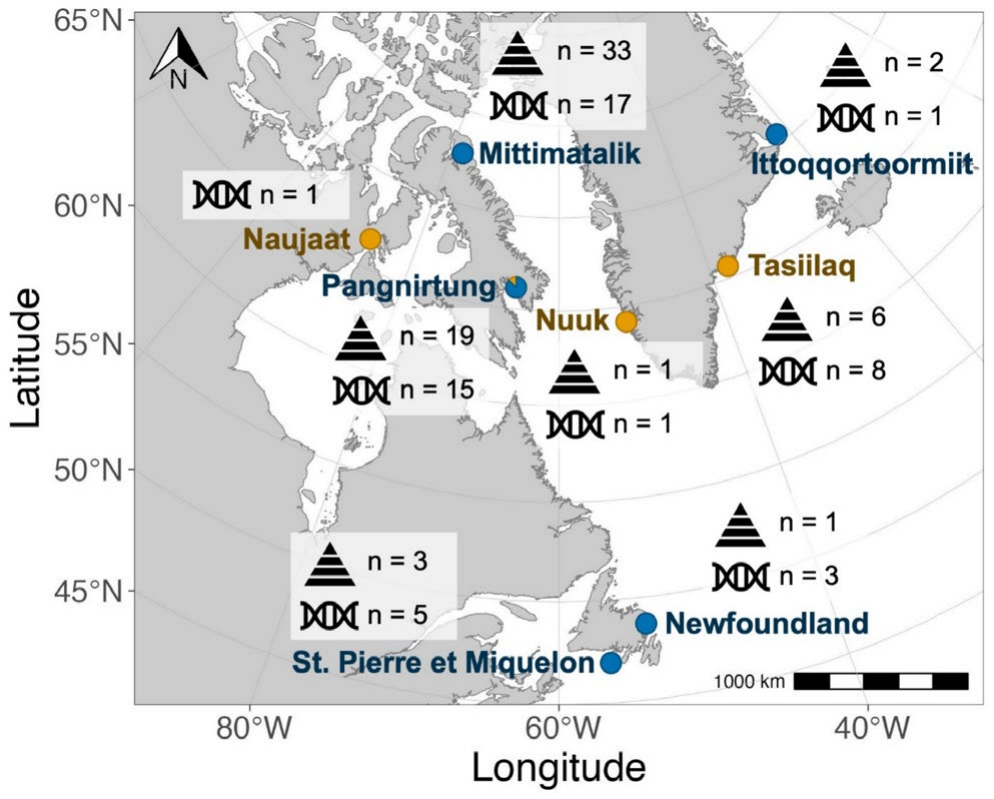
Sampling locations and respective sample size (after removal of duplicates and close kin; see Table S1) in eastern Canada and Greenland where killer whale skin samples were collected for genetic (indicated by DNA icon) and dietary (indicated by trophic triangle icon) analyses between 2008 and 2022. Locations in blue and yellow correspond to genetic populations ECAG1 and ECAG2, respectively.

Skin biopsies were collected from 2013 to 2021 from free‐ranging killer whales during the ice‐free season around Baffin Island near Mittimatalik (Pond Inlet) and Pangnirtung, NU, and further south off the coasts of Newfoundland and St. Pierre et Miquelon (Figure 1). We approached groups of killer whales by boat and sampled juvenile and adult individuals using a crossbow or a CO_2_ rifle equipped with a sterile, tubular steel biopsy tip. Biopsies were collected and immediately frozen in liquid N_2_ cryoshippers. We also received skin samples collected from hunted or stranded killer whales in East (Tasiilaq, 2012; Ittoqqortoormiit, 2021) and West (Nuuk, 2021) Greenland, Newfoundland (2008, 2019), Naujaat, NU (2009), and Mittimatalik (2022, Figure 1). All samples (Table S1) were frozen and shipped to the Freshwater Institute in Winnipeg, Manitoba, where they were stored at −80°C.

### 2.2 DNA Extraction and Whole Genome Sequencing (WGS)

DNA was extracted from subsamples of skin (*n* = 91; Table S1) and sent to the Centre for Applied Genomics at SickKids Hospital in Toronto, ON, for whole genome sequencing. We prepared sequence data for analysis following Garroway et al. (2024); Supporting Information. Briefly, we trimmed and mapped reads to a reference genome (accession #GCA_937001465.1, Foote et al. 2022). We downsampled genomes to avoid imbalances in read coverage before calling genetic variants, including single nucleotide polymorphisms (SNPs). We identified resampled individuals and close kin using kinship coefficients ($\hat{\pi }$), and removed genetic duplicates and first‐degree relatives in ECAG1 ($\hat{\pi }$ > 0.45). Since ECAG2 is highly inbred (all individuals $\hat{\pi }$ > 0.44), we did not remove close kin in this population. The final WGS sample size was 51 after all removals (Table S1). To prepare the genomic dataset for analysis, we filtered SNPs to remove low‐quality sites, sites with high missingness, small scaffolds, sex‐linked SNPs, sites out of Hardy–Weinberg equilibrium, and pruned sites for linkage disequilibrium. Finally, given that bulk isotope composition can differ between sexes in killer whales (e.g., Samarra et al. 2017), we determined sex for each individual (Bérubé and Palsbøll 1996; Smith et al. 2018).

### 2.3 Compound‐Specific Stable Isotope Analysis (CSIA‐AA)

Skin subsamples (*n* = 81; Table S1) were finely diced, freeze‐dried for 48 h, and homogenized using a mortar and pestle. Lipids were extracted by adding 1.9 mL 2:1 chloroform:methanol to each sample, vortexing, and placing the samples in a 30°C water bath for 24 h. Solvents were removed from the vial and the process was repeated once before drying the samples for 24 h. CSIA‐AA of each sample was conducted at the Stable Isotope Facility at the University of California, Davis, following the protocol described in detail by Matthews et al. (2024). Briefly, samples were hydrolysed in HCl under a N_2_ headspace before δ^13^C and δ^15^N measurement of AAs derivatized as N‐acetyl methyl esters (δ^13^C of all samples except 2013 and δ^15^N of all samples; Corr et al. 2007) or using methoxycarbonylation (δ^13^C 2013 samples; Walsh et al. 2014). AA derivatives were separated and measured using GC‐C‐IRMS on a gas chromatograph coupled to an isotope ratio mass spectrometer. δ^13^C and δ^15^N were measured in 12 AAs: alanine (Ala), aspartic acid + asparagine (Asx), glutamic acid + glutamine (Glx), glycine (Gly), isoleucine (Ile), leucine (Leu), lysine (Lys), methionine (Met), phenylalanine (Phe), proline (Pro), valine (Val), and threonine (Thr). Calibration, scale‐normalization, and quality control followed Yarnes and Herszage (2017). Laboratory reference materials were measured in replicate every five samples, and all samples were analyzed in duplicate or triplicate. Mean standard deviations (across all AAs) of replicate sample measurements were ±0.26‰ for δ^13^C and ±0.36‰ for δ^15^N, and were ±0.84‰ for δ^13^C and ±0.95‰ for δ^15^N for replicate reference material measurements.

### 2.4 Analysis

#### 2.4.1 Genetic Population Structure

We used the R package pcadapt 4.3.5 (Privé et al. 2020) to assess killer whale population structure using a principal component analysis (PCA). For comparison, we also ran a PCA for the set of genomes with no kin removed from either population. We estimated genetic differentiation between the groups identified in the PCA by calculating the fixation index (*F*_ST_), which relates the genetic variation within a population to the total genetic variation among populations using the R package StAMPP 1.6.3 (Pembleton et al. 2013). *F*_ST_ is bounded between 0 and 1, with *F*_ST_ values between 0.15 and 0.30 generally considered high, indicating little to no gene flow between populations (Holsinger and Weir 2009; Pilot et al. 2010). We used the groups identified by PCA for subsequent analysis of the CSIA‐AA data.

#### 2.4.2 Linear Discriminant Analysis of Amino Acid δ^13^C and δ^15^N

The carbon (δ^13^C) and nitrogen (δ^15^N) stable isotope ratios of individual amino acids (AAs) reflect unique metabolic pathways that allow for differentiation among confounding ecological factors—namely distribution/habitat use and diet—and thus for more detailed inferences than bulk tissue stable isotope analysis (Whiteman et al. 2019). Essential amino acids (AA_ESS_) cannot be synthesized by vertebrate consumers, so δ^13^C values set by primary producers are largely conserved throughout food webs, providing a proxy for spatial distribution or foraging habitat (both of which can vary considerably in baseline δ^13^C values). AA‐specific δ^15^N differs largely between two classes of AAs: “trophic” AAs that undergo deamination and transamination reactions with associated isotopic fractionation that leads to ^15^N enrichment with each trophic level, and “source” AAs whose amine bond is not broken during typical metabolism and thus retain the approximate baseline δ^15^N values of the food web (similarly to, but independent of, AA_ESS_ δ^13^C). In addition to baseline inferences made from source δ^15^N variation, trophic inferences can be made by calibrating trophic AA δ^15^N against that of source AAs (further division of this difference by the appropriate trophic discrimination factor, which is not well‐resolved for killer whales, would allow for estimation of absolute trophic level). In these ways, AA‐specific δ^13^C and δ^15^N can discern between the two key ecological contributors—diet and distribution or habitat use—to bulk SI variation of consumer tissues (McClelland and Montoya 2002; Chikaraishi et al. 2007, 2009). Here, we use CSIA‐AA of δ^13^C and δ^15^N as indicators of distribution/habitat use and diet, respectively, to examine ecological differences among genetic populations.

We used linear discriminant analysis (LDA) with R package MASS (Venables and Ripley 2002), which reduces the dimensionality of multivariate data by finding linear features that maximize variance between labeled classes, to evaluate ecological differences between genetic populations based on AA‐specific SI proxies. The LDA included δ^13^C values of five AA_ESS_ (Ile, Leu, Met, Phe, and Val; McMahon et al. 2010; Matthews et al. 2024), δ^15^N of two trophic‐source AA pairings (Glx‐Phe, Thr‐Phe), and δ^15^N of source AA Lys (Table S2). δ^15^N_Glx‐Phe_ is the most common trophic‐source pairing used to evaluate consumer trophic level (McMahon and McCarthy 2016), and has been shown to be *negatively*, rather than positively, correlated with trophic level in killer whales (Matthews et al. 2024). Of all AAs, δ^15^N_Thr_ exhibits the strongest linear correlation with trophic level (Bradley et al. 2015), and both δ^15^N_Thr_ and δ^15^N_Thr‐Phe_ are negatively correlated with known trophic differences in fish‐ and mammal‐eating killer whales (Matthews et al. 2024). Source AAs δ^15^N_Phe_ and δ^15^N_Lys_ provide an additional proxy of spatial distribution (McMahon et al. 2015). δ^15^N of trophic AA Thr and source AA Phe, however, were excluded from the LDA due to collinearity with δ^15^N_Thr‐Phe_ and δ^15^N_Glx‐Phe_, respectively. Duplicate samples collected in the same or different years (determined from WGS) were excluded from the LDA and subsequent CSIA‐AA analyses (*n* = 65 after removal of duplicates; Table S1).

#### 2.4.3 Amino Acid‐Specific δ^13^C and δ^15^N

Following the LDA, we tested whether δ^13^C of the five AA_ESS_ differed individually between genetic populations using multivariate linear regression in the R package brms (Bürkner 2017). We used univariate linear regressions in brms to determine whether trophic‐source AAs δ^15^N_Glx‐Phe_ and δ^15^N_Thr‐Phe_, trophic AA δ^15^N_Thr_, and source AAs δ^15^N_Phe_ and δ^15^N_Lys_ differed between genetic populations.

Isotopic turnover rates of δ^13^C and δ^15^N in the skin of common bottlenose dolphins ( *Tursiops truncatus* ), another delphinid, are estimated to have a mean half‐life of 11–48 days (Browning et al. 2013; Giménez et al. 2016) and thus reflect the isotopic composition of their prey in the weeks to months leading up to sampling. Since killer whales are highly mobile, capable of traveling hundreds of kilometers in several days or weeks (Matthews et al. 2011), it is unlikely that isotopic values are representative of where the individual was sampled, especially in Arctic locations where killer whales are only seasonally resident (Matthews et al. 2024). We therefore did not include sample location in models of SI variation, and instead infer potential spatial foraging patterns from variation in AA‐specific δ^13^C and δ^15^N, which reflect isotopic baselines. Most killer whales were sampled in August and early September. However, a few individuals were sampled off St. Pierre et Miquelon (*n* = 3) and Nuuk, West Greenland (*n* = 1) in June and November, respectively, a period during which measurable isotopic turnover can occur. To ensure that CSIA‐AA values were comparable between sexes and across the months in which sampling occurred, we tested whether δ^13^C of the five AA_ESS_, δ^15^N_Glx‐Phe_, δ^15^N_Thr_, and δ^15^N_Thr‐Phe_ differed with sex or day of year modeled as covariates with genetic population. All models were run with default uninformative priors and three parallel chains of 50,000 draws with a burn‐in of 20,000 draws. Model chain convergence was visually assessed using parameter trace plots and density plots, and by ensuring the Gelman‐Rubin statistic was below 1.1 (Gelman and Rubin 1992). We evaluated model fit using graphical posterior predictive checks (Gabry et al. 2019).

#### 2.4.4 Individual Variation in AA δ^13^C and δ^15^N

Seven killer whales from the ECAG1 population were resampled in different years, providing the opportunity to examine individual isotopic variation. To compare individual δ^13^C AA_ESS_ values across different sampling occasions, we ran a PCA including resampled individuals along with those in their sample groups (e.g., same location and year). We also calculated the difference in δ^15^N_Glx‐Phe_, δ^15^N_Thr_, and δ^15^N_Thr‐Phe_ values of resampled individuals between sampling occasions. All analyses in R were performed in v4.4.1 (R Core Team 2024).

## 3 Results

### 3.1 Genetic Population Structure

**FIGURE 2:**
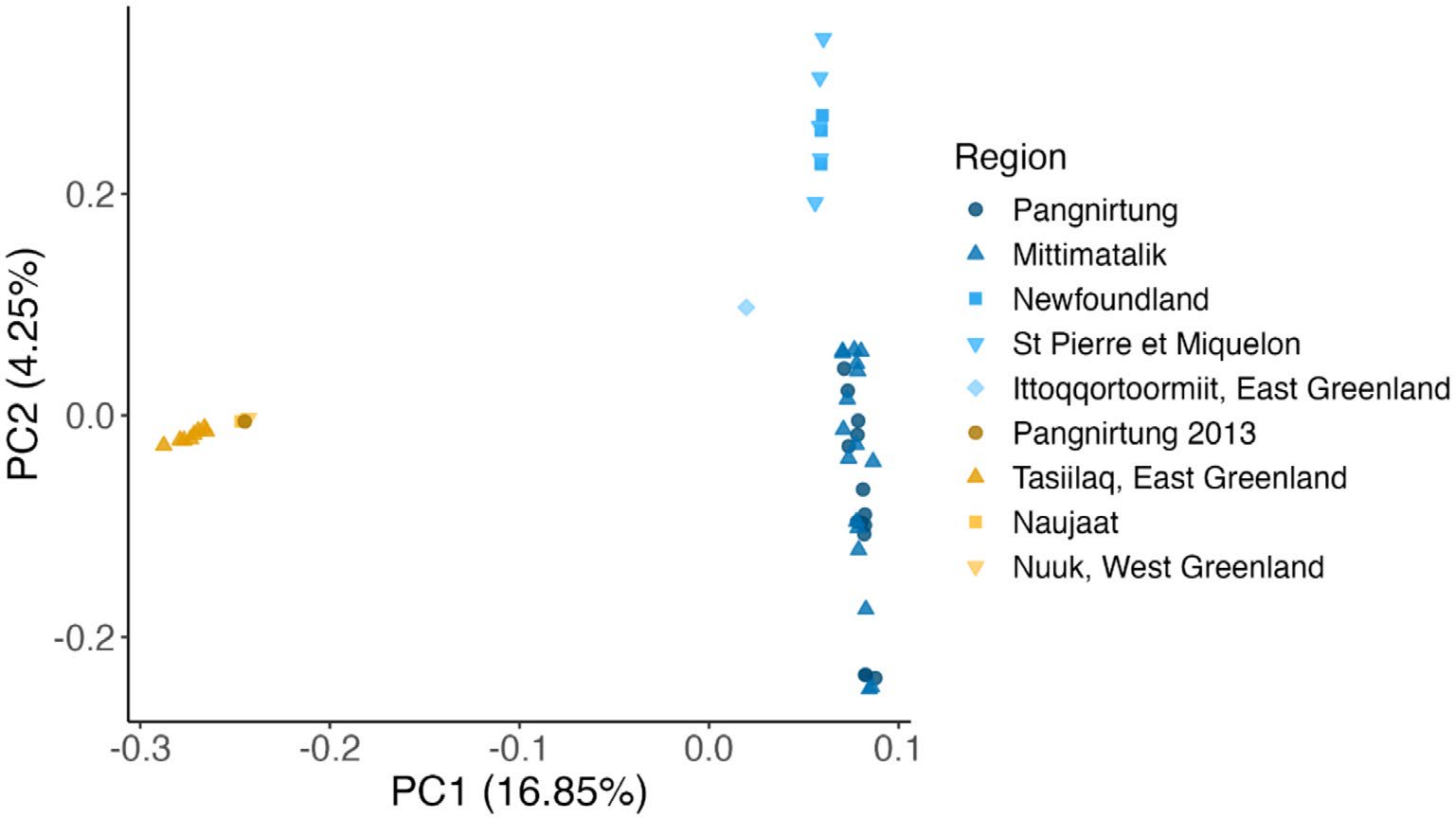
Principal component analysis of whole genome sequence data showing the genetic population structure of killer whales (*n* = 51) in the eastern Canadian Arctic and Greenland. Sampling locations corresponding to genetic populations ECAG1 and ECAG2 are indicated in blue and yellow, respectively.

The PCA of SNP data showed two primary distinct clusters, separating a large group of individuals corresponding to ECAG1 and a smaller group of individuals corresponding to ECAG2 along the PC1 axis (Figure 2). The ECAG2 cluster included individuals sampled near Tasiilaq (East Greenland), Nuuk (West Greenland), Naujaat, and one individual sampled near Pangnirtung in 2013. The substantial proportion of genetic variation explained by PC1 (16.85%) was supported by high values of differentiation between ECAG1 and ECAG2 (*F*_ST_ = 0.225, 95% CI = 0.225–0.226). The inclusion of ECAG1 kin in the PCA did not substantially influence the segregation of ECAG1 and ECAG2 along PC1, or the structure of ECAG2 along either axis (Figure S1A).

The PCA also revealed substructure within ECAG1, with separation of the individual sampled near Ittoqqortoormiit (East Greenland) and those sampled near St. Pierre et Miquelon and Newfoundland from those sampled near Mittimatalik and Pangnirtung along PC2 (explaining 4.25% of the variation; Figure 2). *F*_st_ values corroborated that the genetic subcluster of individuals sampled near Mittimatalik and Pangnirtung was differentiated, to a lesser degree compared to ECAG1 and ECAG2, from the subcluster of individuals sampled near St. Pierre et Miquelon and Newfoundland (*F*_ST_ = 0.042, 95% CI = 0.042–0.042; Figure 1; for PCA of ECAG1 only see Figure S1B).

### 3.2 Linear Discriminant Analysis of AA δ^13^C and δ^15^N

**FIGURE 3:**
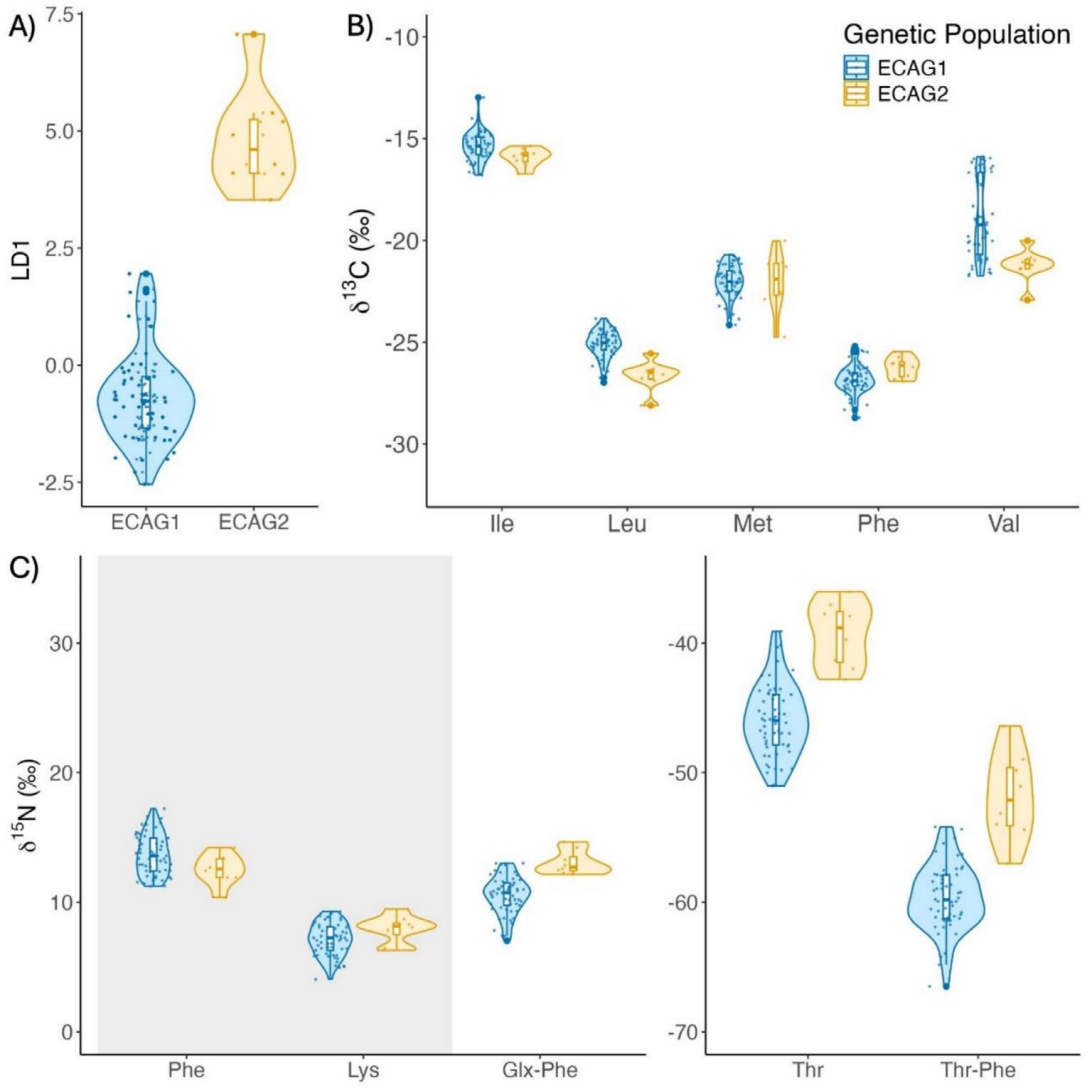
Comparisons of compound‐specific stable isotope values between killer whale populations ECAG1 (blue) and ECAG2 (yellow) in the northwest Atlantic. (A) Linear discriminant analysis showed separation between ECAG1 and ECAG2 AA‐specific δ^13^C and δ^15^N values. (B) δ^13^C values for four of five AA_ESS_ (Ile, Leu, Phe, Val) differed between populations. (C) Paired trophic‐source AA δ^15^N_Glx‐Phe_ and δ^15^N_Thr‐Phe_ and trophic AA δ^15^N_Thr_ values differed between populations, while source AA δ^15^N_Phe_ and δ^15^N_Lys_ values (highlighted in gray) did not.

LDA classified all 65 samples to the expected genetic population, with differences between populations primarily driven by δ^13^C_Leu_ and δ^15^Ν_Thr‐Phe_, (coefficients of LD1 = −1.48 and 0.78, respectively), followed by δ^13^C_Phe_ (0.61), δ^15^Ν_Glx‐Phe_ (0.44), δ^13^C_Lys_ (0.37), δ^13^C_Ile_ (0.29), δ^13^C_Met_ (0.22), and δ^13^C_Val_ (0.08; Figure 3A).

### 3.3 Amino Acid‐Specific δ^13^C and δ^15^N

Multivariate regression revealed ECAG1 had significantly higher δ^13^C_Ile_, δ^13^C_Leu_, and δ^13^C_Val_ and lower δ^13^C_Phe_ than ECAG2, while δ^13^C_Met_ did not differ (Figure 3B; Tables 1 and 2). ECAG1 had significantly lower δ^15^N_Glx‐Phe_, δ^15^N_Thr_, and δ^15^N_Thr‐Phe_ than ECAG2 (Figure 3C; Tables 1 and 2). δ^15^N of source amino acids Phe and Lys did not differ between the two genetic populations (Figure 3C; Tables 1 and 2). No differences in any modeled parameter (δ^13^C AA_ESS_, δ^15^N_Glx‐Phe_, δ^15^N_Thr_, and δ^15^N_Thr‐Phe_) with sex (Figure S2, Table S3) or day of year (Figure S3, Table S4) were detected.

**TABLE 1:** Mean ± standard error of amino acid (AA)‐specific δ^13^C for five essential amino acids (Ile, Leu, Met, Phe, and Val), and δ^15^N of two trophic‐source AA pairings (Glx‐Phe, Thr‐Phe), a trophic AA (Thr), and two source AAs (Phe, Lys) for killer whale populations ECAG1 (*n* = 57) and ECAG2 (*n* = 8) in the northwest Atlantic.

δ^13^C_AA_	ECAG1	ECAG2	δ^15^N_AA_	ECAG1	ECAG2
Ile	−15.38‰ ± 0.09‰	−15.92‰ ± 0.16‰	Glx‐Phe	10.53‰ ± 0.19‰	13.06‰ ± 0.33‰
Leu	−25.09‰ ± 0.09‰	−26.63‰ ± 0.26‰	Thr	−45.96‰ ± 0.36‰	−39.32‰ ± 0.88‰
Met	−21.99‰ ± 0.10‰	−22.04‰ ± 0.52‰	Thr‐Phe	−59.65‰ ± 0.33‰	−51.85‰ ± 1.21‰
Phe	−26.83‰ ± 0.09‰	−26.24‰ ± 0.18‰	Phe	13.68‰ ± 0.21‰	12.53‰ ± 0.42‰
Val	−18.90‰ ± 0.26‰	−21.23‰ ± 0.29‰	Lys	7.12‰ ± 0.16‰	7.93‰ ± 0.38‰

**TABLE 2:** Results from multivariate (δ^13^C of essential amino acids Ile, Leu, Met, Phe, and Val) and univariate (δ^15^N of trophic and source amino acids Glx‐Phe, Thr, Thr‐Phe, Phe, and Lys) models examining differences between genetic populations ECAG1 and ECAG2, measured in killer whale skin samples collected across the northwest Atlantic (*n* = 65).

Model	Coefficients	Estimate	Est. error	L‐95% CI	U‐95% CI
δ^13^C AA_ESS_~genetic population	Ile intercept	−15.38	0.09	−15.56	−15.19
	Leu intercept	−25.09	0.09	−25.27	−24.91
	Met intercept	−21.99	0.12	−22.22	−21.75
	Phe intercept	−26.83	0.09	−27.01	−26.65
	Val intercept	−18.91	0.26	−19.41	−18.40
	*Ile genetic population ECAG2*	−0.54	0.27	−1.06	−0.02*
	*Leu genetic population ECAG2*	−1.54	0.26	−2.05	−1.03*
	Met genetic population ECAG2	−0.05	0.34	−0.72	0.62
	*Phe genetic population ECAG2*	0.59	0.26	0.09	1.10*
	*Val genetic population ECAG2*	−2.33	0.73	−3.77	−0.89*
δ^15^N_Glx‐Phe_~genetic population	Intercept	10.53	0.19	10.16	10.89
	*Genetic population ECAG2*	*2.53*	*0.54*	*1.48*	3.58*
δ^15^N_Thr_~genetic population	Intercept	−45.97	0.36	−46.69	−45.25
	*Genetic population ECAG2*	*6.64*	*1.04*	*4.58*	8.68*
δ^15^N_Thr‐Phe_~genetic population	Intercept	−59.65	0.35	−60.35	−58.96
	*Genetic population ECAG2*	*7.80*	*1.01*	*5.81*	9.78*
δ^15^N_Phe_~genetic population	Intercept	13.68	0.20	13.28	14.08
	Genetic population ECAG2	−1.15	0.58	−2.30	0.00
δ^15^N_Lys_~genetic population	Intercept	7.13	0.16	6.81	7.45
	Genetic population ECAG2	0.80	0.47	−0.11	1.73

*Note:* For each AA or paired AA examined, ECAG2 was interpreted to significantly differ (indicated by italics and an asterisk) from ECAG1 when the 95% Bayesian credible interval (L = lower limit, U = upper limit) for ECAG2 did not overlap with zero.

The Gelman‐Rubin diagnostic (< 1.01), parameter trace plots, and density plots indicated convergence for all models, and posterior predictive plots indicated adequate model performance (Figure S5A,B). Due to small sample size, we did not test for differences in δ^13^C AA_ESS_ or δ^15^N AA between the ECAG1 subclusters (Mittimatalik/Pangnirtung and Newfoundland/St. Pierre et Miquelon; Figure S4).

### 3.4 Individual Variation in AA δ^13^C and δ^15^N

**FIGURE 4:**
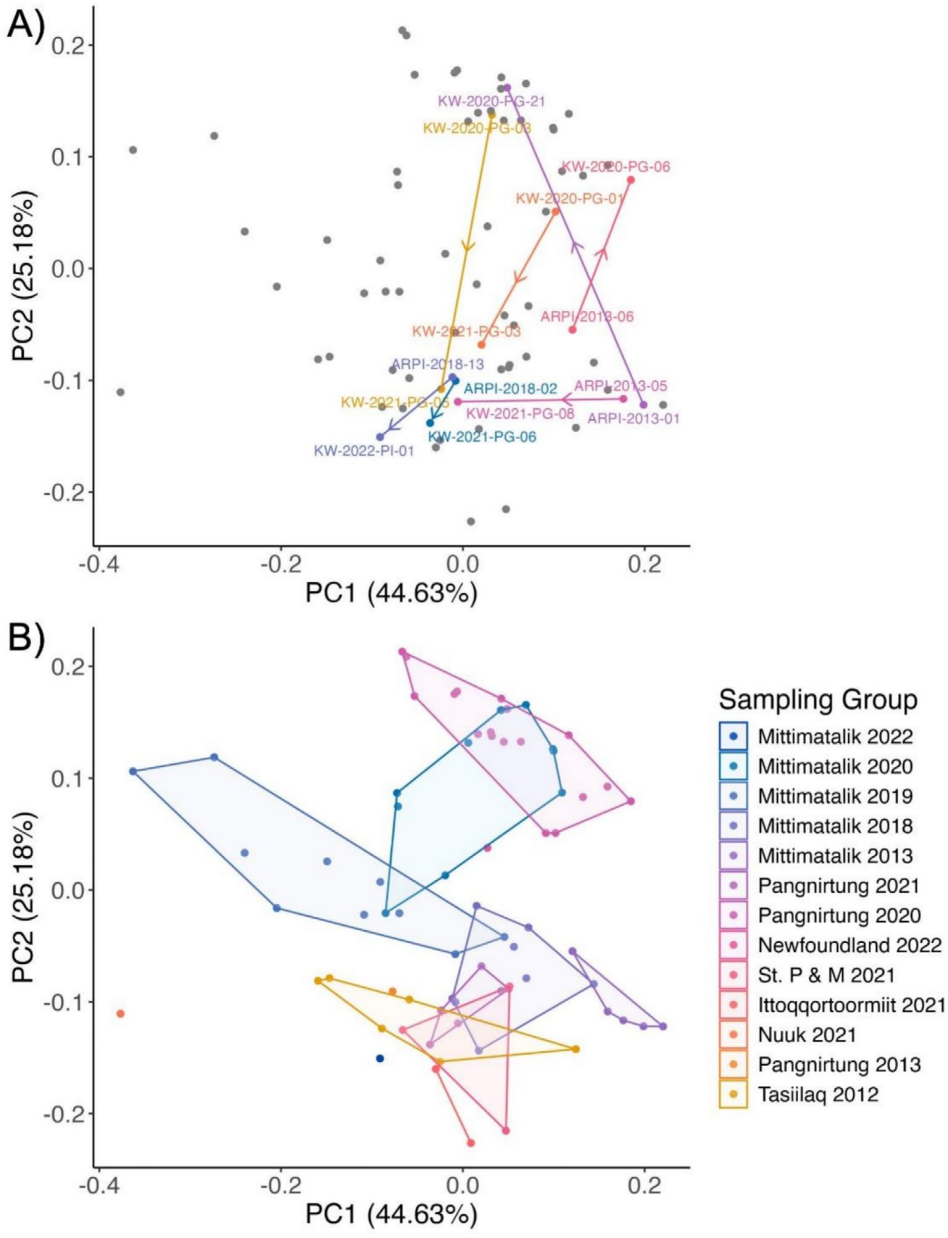
Principal component analysis of δ^13^C values for five essential amino acids (AA_ESS_; Ile, Leu, Met, Phe, Val) in skin biopsies from killer whales in the eastern Canadian Arctic. Panel (A) shows seven individuals that were resampled in different years (total *n* = 72), with solid lines connecting the values of the same individual on two different sampling occasions and the year of sample indicated in the sample name label. Arrows indicate direction from earlier to later samples. Panel (B) shows groups sampled in each location and year.

δ^13^C AA_ESS_ values of resampled killer whales were more similar to those of other killer whales they were sampled with (same location and year) than to their own values from different sampling years (Figure 4A,B). For example, the groups sampled near Pangnirtung in 2020 and 2021 cluster at opposite ends of PC2 but include individuals that were sampled on both occasions (e.g., KW‐2020‐PG‐03/KW‐2021‐PG‐05).

**FIGURE 5:**
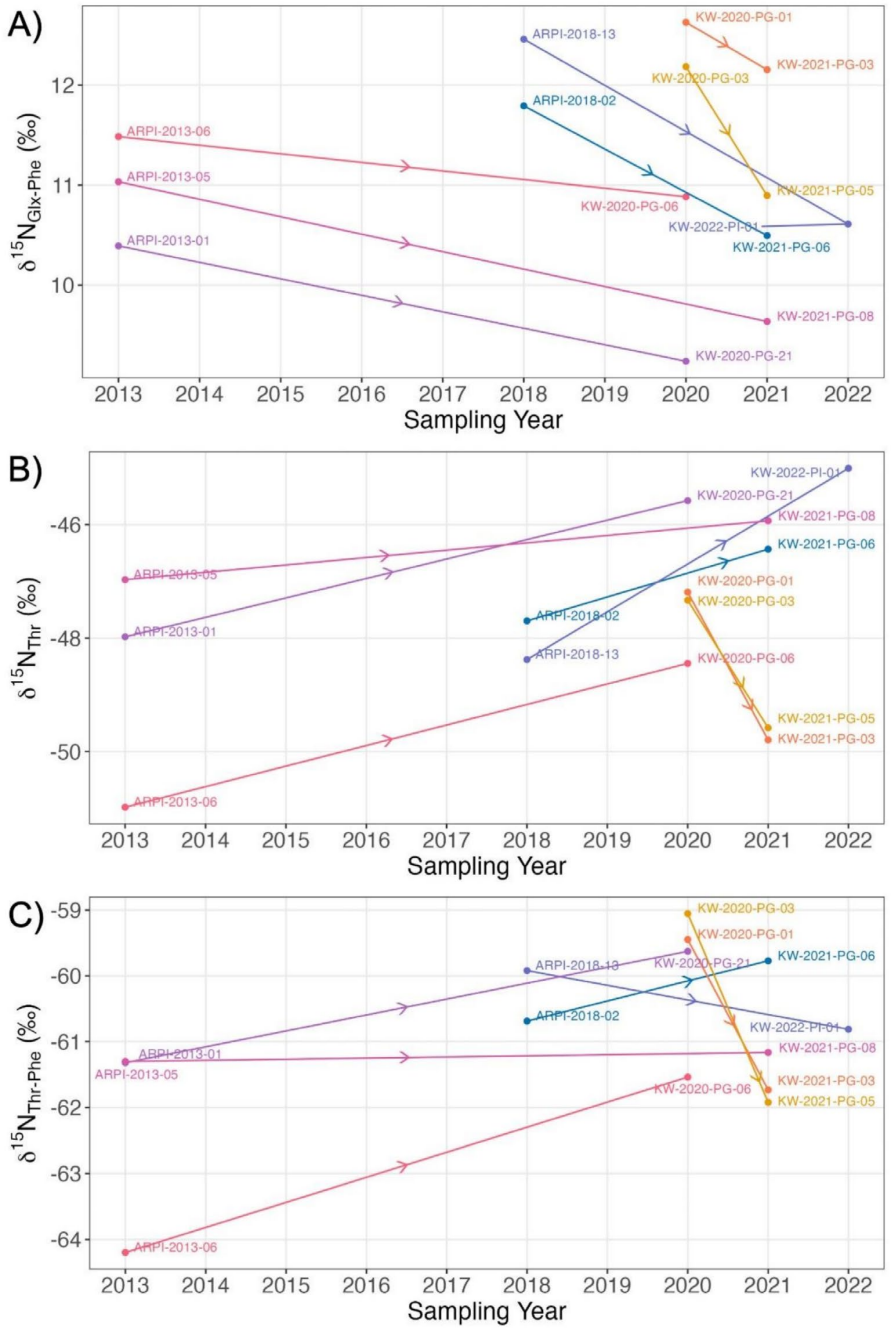
Skin (A) δ^15^N_Glx‐Phe_, (B) δ^15^N_Thr_, and (C) δ^15^N_Thr‐Phe_ values for seven resampled killer whales, with solid lines connecting samples from the same individual in different sampling years. Arrows indicate direction from earlier to later sample.

When comparing the δ^15^N AA values of individuals resampled in different years, δ^15^N_Glx‐Phe_ decreased for all individuals between the first and second sampling occasion (min difference = −1.85‰, max difference = −0.48‰; Figure 5A). δ^15^N_Thr_ and δ^15^N_Thr‐Phe_ did not exhibit consistent patterns across all resampled whales, with measurements increasing between the first and second sampling occasions in some individuals (δ^15^N_Thr_: 5 of 7, δ^15^N_Thr‐Phe_: 4 of 7), and decreasing in others (δ^15^N_Thr_: 2 of 7, min = −2.61‰, max = 3.37‰; δ^15^N_Thr‐Phe_: 3 of 7, min = −2.87‰, max = 2.66‰; Figure 5B,C).

## 4 Discussion

Ecological characterization of two genetically distinct populations in the northwest Atlantic and Greenland, using stable isotope analysis, indicates they are also differentiated by their distribution or habitat use patterns and by their diet. We demonstrate concurrent genetic and ecological (spatial and dietary) variation, comparable to population‐level variation observed in other regions; for example, in the North Pacific. Within the context of their summer presence in the Arctic, the differentiation of these populations poses important questions about their ecological influence, particularly on the marine mammals that dominate their diet (Remili et al. 2023; Matthews et al. 2024), and evolutionary trajectory under current and future range expansion in the Arctic.

The overall patterns of genomic population structure exhibited two highly distinct genetic populations that aligned with ECAG1 and ECAG2 proposed by Garroway et al. (2024). The high *F*_ST_ of 0.23 indicates substantial genetic differentiation between populations with little to no gene flow, comparable to the difference between subspecies and ecotypes in the North Pacific (pairwise *F*_ST_ for eight North Pacific populations = 0.04–0.24, Hoelzel et al. 2007; Pilot et al. 2010). The clustering of killer whale samples from East and West Greenland with both ECAG1 and ECAG2 supports previously proposed connections between the eastern Canadian Arctic and Greenland (Reeves and Mitchell 1988; Matthews et al. 2024). The addition of new samples also provided evidence for substructure within ECAG1, indicating there may be further levels of genetic divergence in northwest Atlantic killer whales. In particular, the differentiation between individuals sampled near Mittimatalik and Pangnirtung and those sampled near Newfoundland and St. Pierre et Miquelon suggests further segregation within genetic clusters. Additional sampling, including in other areas of the eastern Canadian Arctic (e.g., Hudson Bay), will help to clarify the degree of genetic differentiation both between and within populations.

Our updated analysis included 13 additional samples from Pangnirtung (*n* = 23, including genetic duplicates and close kin; see Table S1). Unlike the single Pangnirtung sample from 2013 included in Garroway et al. (2024) that clustered with ECAG2, the new samples clustered with ECAG1, indicating they belong to the same genetic population as those sampled near Mittimatalik. This is consistent with the re‐sampling of four individuals biopsied near both Pangnirtung and Mittimatalik and the re‐sightings of photo‐identified individuals between Pangnirtung and Mittimatalik (Kucheravy et al. 2023), as well as the general movements of killer whales with receding sea ice northward along the east coast of Baffin Island in spring (Lefort et al. 2020). The Pangnirtung 2013 sample, which was the lone whale sampled from its group, groups clearly with ECAG2 in both the genetic (Figure 2) and CSIA‐AA (Figure 3, Table S2) analyses suggesting that it is not an outlier in the population, but rather indicative of sympatry between the two populations. The δ^13^C AA_ESS_ results and spatial distribution of sampling locations corresponding to the two populations (Figure 1, Garroway et al. 2024) also suggest there is some geographic overlap between ECAG1 and ECAG2. While individuals from both populations may visit Cumberland Sound, killer whales from ECAG1 may be more abundant in that area and therefore more likely to be sampled.

Minimal overlap of AA‐specific δ^13^C and δ^15^N, proxies for distribution or habitat use and diet, indicates ecological differentiation between the two populations. Evidence of different spatial distributions was supported by the LDA and significant differences in four of the five δ^13^C AA_ESS_ (Ile, Leu, Phe, Val) between ECAG1 and ECAG2 in the multivariate model. Conversely, δ^15^N of the source AAs Phe and Lys, which are assumed to reflect regional baselines (McClelland and Montoya 2002; Chikaraishi et al. 2007, 2009), did not differ between the two populations. The two populations may range over areas that differ in baseline δ^13^C but not in δ^15^N. Isoscapes of primary producer bulk δ^13^C and δ^15^N can be broadly similar over vast oceanic regions in the mid‐ and North Atlantic (e.g., similar δ^13^C values from the east of Newfoundland to south of Greenland; Magozzi et al. 2017), while regional differences in baseline δ^13^C and δ^15^N are not necessarily spatially aligned (Graham et al. 2010; McMahon et al. 2013). Further, δ^15^N_Phe_ undergoes marginal trophic enrichment of approximately 0.4‰ (Chikaraishi et al. 2009) with evidence of greater enrichment under some circumstances (Nuche‐Pascual et al. 2018; Matthews et al. 2020). While small, cumulative Phe ^15^N enrichment could be large enough in high trophic level organisms, such as killer whales, to obscure baseline variation. Despite significant differences, overlap in δ^13^C AA_ESS_ values indicates the populations may occupy neighboring or partially sympatric geographical ranges in the northwest Atlantic. Significant differences in δ^13^C AA_ESS_ could also be indicative of smaller scale habitat differences (e.g., between nearshore and offshore habitats; Larsen et al. 2020; Stahl et al. 2023) within a largely homogenous δ^15^N isoscape. This would be comparable to the distributions of North Pacific killer whales, where the “Resident” (primarily coastal/nearshore) and “Offshore” killer whale populations, for example, overlap in range but differ in general habitat and movement patterns (Dahlheim et al. 2008; Morin et al. 2024). Different distributions inferred here are consistent with isotope‐based (δ^18^O and AA‐specific δ^15^N) studies of teeth from other Arctic killer whales that suggest variable distributions within a broader North Atlantic range (Matthews and Ferguson 2014; Matthews, Longstaffe, et al. 2021; Matthews et al. 2024).

The consistently lower δ^15^N_Glx‐Phe_, δ^15^N_Thr_, and δ^15^N_Thr‐Phe_ values of ECAG1 relative to ECAG2 also indicate ecologically relevant trophic differences between the two populations. Estimating absolute trophic differences from AA‐specific δ^15^N, as with bulk δ^15^N, requires accurate trophic discrimination factors (TDF), which are the differences in stable isotope values between the consumer and its diet (Post 2002). TDF_Glx‐Phe_ is variable in marine food webs (McMahon and McCarthy 2016), especially among upper trophic level marine consumers (Germain et al. 2013; Matthews et al. 2020). In killer whales, the relative difference in δ^15^N values between trophic‐source AA pairings decreases with higher trophic levels, rather than increases as expected from studies of organisms at lower trophic positions (Matthews, Lawson, and Ferguson 2021; Matthews et al. 2024). Relying therefore more simply on ΑΑ−specific δ^15^N values as a relative index of trophic position, the lower δ^15^N_Glx‐Phe_, δ^15^N_Thr_, and δ^15^N_Thr‐Phe_ values indicate ECAG1 killer whales feed at a higher trophic level than ECAG2. For context, the ~2.4‰ difference in mean δ^15^N_Glx‐Phe_ values between ECAG1 and ECAG2 is comparable to the trophic step calculated for mammal‐eating killer whales using δ^15^N_Glx‐Phe_ values from dentin collagen (~2.2‰ to 2.9‰; Matthews et al. 2020). The differences in mean δ^15^N_Thr_ and δ^15^N_Thr‐Phe_ values between ECAG1 and ECAG2 killer whales were approximately −6.8‰ and −7.9‰, similar to those between killer whale ecotypes with known marine mammal or fish diets (Matthews et al. 2024), as well as the −6‰ difference in δ^15^N_Thr_ calculated with each trophic step for other vertebrate consumers (Hare et al. 1991; Styring et al. 2010; Bradley et al. 2015). These differences are consistent with reported killer whale diets in the eastern Canadian Arctic and Greenland (Heide‐Jørgensen 1988; Ferguson et al. 2012; Foote et al. 2013; Remili et al. 2023), and with the range of mammal‐based, fish‐based, and mixed diets described for other North Atlantic killer whale populations (Foote et al. 2012; Jourdain et al. 2020; Remili et al. 2023).

In addition to population‐level differentiation, the differences in individual δ^15^N AA and δ^13^C AA_ESS_ values between sampling occasions indicate considerable flexibility in both distribution and diet. In the eastern Canadian Arctic, killer whales have been observed eating Arctic‐endemic whales and seals (Ferguson et al. 2012; Higdon et al. 2012), but their diet appears flexible. For example, killer whales around northern Baffin Island consume a greater proportion of narwhal ( *Monodon monoceros* ) than beluga ( *Delphinapterus leucas* ), which are relatively less abundant in that area (Ferguson et al. 2012; Higdon et al. 2012). Similarly, killer whales consume higher proportions of bowhead whales ( *Balaena mysticetus* ) in Cumberland Sound where they are more abundant (Ferguson et al. 2012; Reinhart et al. 2013; Doniol‐Valcroze et al. 2015). The individual variation observed here implies that these killer whales may be equally flexible in the period leading up to their seasonal presence in Arctic waters. During the winter and spring, individuals and/or groups may not return consistently to the same geographic region, potentially altering their movements in response to prey availability (Similä et al. 1996; Nichol and Shackleton 1996) or other environmental variables (Pitman and Ensor 2003; Higdon et al. 2012; Matthews et al. 2019). Interannual variation in distribution inferred from the individuals resampled here is consistent with variation in δ^18^O in annual killer whale dentin growth layer groups, also interpreted by Matthews, Longstaffe, et al. (2021) to indicate individual variation in distribution between years.

Killer whales are summer residents in the Arctic, with an expanding spatial and temporal range corresponding to a lengthening open water season (Higdon and Ferguson 2009; Ferguson et al. 2025; Kimber et al. 2025). As apex predators, killer whales may play an important role in a changing Arctic ecosystem (Ferguson et al. 2010), with their potential top‐down influence depending on their degree of dietary specialization (de Bruyn et al. 2013; Ohlberger et al. 2019; Schmid et al. 2019). Spatial and trophic differentiation between ECAG1 and ECAG2 suggests that the two populations may differ in their regulatory influence on Arctic prey, particularly endemic marine mammal species. Further, generalist traits (Matthews et al. 2024) and the plasticity inferred here will likely be beneficial for further range expansion of killer whales in the Arctic (Donelson et al. 2019). Whereas some killer whale populations have a relatively narrow range and diet in some regions (Ford et al. 1998; Hauser et al. 2007; Fearnbach et al. 2014) and therefore presumably more vulnerable to changing conditions (Ford et al. 2010), ecological flexibility will likely favor not only spatial expansion in the Arctic (Monaco et al. 2020; Lanszki et al. 2022; Matthews et al. 2024), but also dietary adaptability to climate‐induced changes to community structure (Fossheim et al. 2015).

From an evolutionary standpoint, the genetic variation observed between the two populations likely developed in isolation (Bolnick and Fitzpatrick 2007; Foote and Morin 2015). ECAG1 and ECAG2 are estimated to have diverged 9–20 thousand years ago (Garroway et al. 2024), likely in allopatry under different ecological conditions near the last glacial maximum, and are now experiencing secondary contact. Based on genetic evidence thus far, they remain reproductively isolated despite spatial proximity in the northwest Atlantic, possibly due to genetic or behavioral barriers. Further spatial and temporal convergence within an expanding Arctic range raises questions about their future evolutionary trajectory. Given the ecological plasticity shown here, the dietary and spatial differentiation between populations may eventually change as these killer whales adapt to common habitat and resources in the Arctic. While the genetic difference between the two populations is currently substantial, this level of differentiation could be lost quickly if ecological factors important for maintaining reproductive isolation are altered due to ongoing climate change. Alternatively, the maintenance of genetic difference between populations despite ecological change could progress toward speciation, as is proposed in other killer whale populations.

## Author Contributions

**Caila E. Kucheravy:** conceptualization (equal), data curation (equal), formal analysis (lead), funding acquisition (supporting), investigation (lead), visualization (lead), writing – original draft (lead), writing – review and editing (lead). **Evelien de Greef:** data curation (equal), formal analysis (supporting), visualization (supporting), writing – review and editing (supporting). **Steven H. Ferguson:** conceptualization (equal), funding acquisition (equal), resources (equal), writing – review and editing (supporting). **Jack W. Lawson:** funding acquisition (equal), resources (equal), writing – review and editing (supporting). **Cortney A. Watt:** funding acquisition (equal), resources (equal), writing – review and editing (supporting). **Jeremy J. Kiszka:** funding acquisition (equal), resources (equal), writing – review and editing (supporting). **Colin J. Garroway:** conceptualization (equal), funding acquisition (equal), resources (equal), writing – original draft (supporting), writing – review and editing (supporting). **Cory J. D. Matthews:** conceptualization (equal), funding acquisition (equal), resources (equal), writing – original draft (supporting), writing – review and editing (supporting).

## Funding

This work was supported by Crownx‐Indigenous Relations and Northern Affairs Canada; Fisheries and Oceans Canada; Nunavut Wildlife Management Board.

## Conflicts of Interest

The authors declare no conflicts of interest.

## Supporting information


**Appendix S1:** ece373593‐sup‐0001‐supinfo.docx.

## Data Availability

Data are publicly available on Dryad at https://doi.org/10.5061/dryad.s1rn8pkpc. Raw genomic data are available on NCBI BioProject PRJNA986581. All relevant code is available at https://github.com/cailakucheravy/kw_differentation_ms.
