# Supplementary material for: Genetic and Ecological Divergence Between Northwest Atlantic Killer Whale Populations
Source: Ecol Evol. 2026 Apr 30;16(5):e73593. doi: 10.1002/ece3.73593 (PMC13130354; doi:10.1002/ece3.73593)
Supplement: Supplementary file 1 — Appendix S1: ece373593‐sup‐0001‐supinfo.docx. [file ECE3-16-e73593-s001.docx]

**Supplemental Material**

**Section A: Supplemental Methods**

*DNA extraction and whole genome sequencing (WGS)*

Subsamples of skin (n = 91) were lysed using Buffer ATL, Proteinase K, and DTT before total genomic DNA was extracted using Qiagen DNeasy blood and tissue kits (Valencia, CA, USA). DNA concentration was measured using a Nanodrop 8000 Spectrophotometer. Whole genomes were sequenced with Illumina sequencing instruments (HiSeq X, NovaSeq 6000, NovaSeq X) by the Centre for Applied Genomics at the SickKids Hospital in Toronto, ON. To prepare sequence data for analysis, reads were trimmed and merged using Trimmomatic 0.35 (Bolger et al. 2014), and then mapped to a chromosome-level reference genome (accession #GCA_937001465.1, Foote et al. 2022) using BWA 0.7.17 (Li and Durbin 2009) and Samtools 1.9 (Li 2011). We removed duplicate reads, added read group information, and filtered the genomes to keep primary alignments using Picard 2.20.6 (Broad Institute 2019) and Samtools 1.9 (Li 2011). To avoid imbalances in read coverage between genomes (9-48x), those exceeding 19x modal coverage were downsampled to 19x using GATK 4.1.2.0 (McKenna et al. 2010). Genetic variants including single nucleotide polymorphisms (SNPs) and insertions and deletions (indels) were called from the processed genomes using Platypus 0.8.1 (Rimmer et al. 2014), while specifying a minimum of four reads reaching default quality thresholds for a variant to be considered.

Re-sampled individuals and close kin were identified from kinship coefficients ($\hat{\pi}$) estimated using plink 1.90 (Purcell et al. 2007) and the package PlinkQC (Meyer 2021), which calculate the proportion of alleles shared at genotyped SNPs to evaluate relatedness in pair-wise comparisons of genomes. We retained one of each duplicate, keeping the sample with the most complete genotype data (lower missingness). In ECAG1, we removed close kin with $\hat{\pi}$ > 0.45 to exclude first-degree relatives. Since ECAG2 is highly inbred (all individuals $\hat{\pi}$ > 0.44), we did not remove close kin in this population. The final WGS sample size was 51 after all removals (Supp. Table 1). To prepare a high quality autosomal genomic dataset for downstream analysis, indels were excluded and SNPs were quality-filtered to remove low-quality sites (QUAL <50, MQ <40, QD <4), sites with high missingness (>0.25 missing site data), small scaffolds (<100kb in length), and sex-linked SNPs using Vcftools 0.1.17 (Danecek et al. 2011) and GATK 4.1.2.0 (McKenna et al. 2010). Finally, sites out of Hardy-Weinberg equilibrium (determined for each population separately) and minor allele frequency < 0.05 were removed, and remaining sites were pruned for linkage disequilibrium (r^2^ > 0.8).

Given that bulk isotope composition can differ between sexes in killer whales (e.g., Samarra et al. 2017), we determined sex for each individual. We examined WGS data using DifCover (Smith et al. 2018) for differences in read coverage for the X chromosome to identify males and females, using a known male genome as a reference. Sex for seven samples that were not whole-genome sequenced was determined using microsatellites by amplifying the ZFX/ZFY region on the sex chromosomes of males and females (Bérubé and Palsbøll 1996).

**Literature Cited:**

Bérubé, M., and Palsbøll, P. 1996. Identification of sex in Cetaceans by multiplexing with three ZFX and ZFY specific primers. Mol. Ecol. **5**(2): 283–287. doi:10.1111/j.1365-294X.1996.tb00315.x.

Bolger, A.M., Lohse, M., and Usadel, B. 2014. Trimmomatic: a flexible trimmer for Illumina sequence data. Bioinformatics **30**(15): 2114–2120. doi:10.1093/bioinformatics/btu170.

Broad Institute. (2019). *Picard Toolkit*. GitHub Repository. https://broadinstitute.github.io/picard/

Danecek, P., Auton, A., Abecasis, G., Albers, C.A., Banks, E., DePristo, M.A., Handsaker, R.E., Lunter, G., Marth, G.T., Sherry, S.T., McVean, G., Durbin, R., and 1000 Genomes Project Analysis Group. 2011. The variant call format and VCFtools. Bioinformatics **27**(15): 2156–2158. doi:10.1093/bioinformatics/btr330.

Foote, A., Bunskoek, P., Wellcome Sanger Institute Tree of Life programme, Wellcome Sanger Institute Scientific Operations: DNA Pipelines collective, Tree of Life Core Informatics collective, and Darwin Tree of Life Consortium. 2022. The genome sequence of the killer whale, *Orcinus* *orca* (Linnaeus, 1758). Wellcome Open Res **7**: 250. doi:10.12688/wellcomeopenres.18278.1.

McKenna, A., Hanna, M., Banks, E., Sivachenko, A., Cibulskis, K., Kernytsky, A., Garimella, K., Altshuler, D., Gabriel, S., Daly, M., and DePristo, M.A. 2010. The Genome Analysis Toolkit: A MapReduce framework for analyzing next-generation DNA sequencing data. Genome Res. **20**(9): 1297–1303. doi:10.1101/gr.107524.110.

Meyer, H. 2021. plinkQC: Genotype Quality Control with “PLINK.” doi:10.32614/CRAN.package.plinkQC.

Li, H. 2011. A statistical framework for SNP calling, mutation discovery, association mapping and population genetical parameter estimation from sequencing data. Bioinformatics **27**(21): 2987–2993. doi:10.1093/bioinformatics/btr509.

Li, H., and Durbin, R. 2009. Fast and accurate short read alignment with Burrows–Wheeler transform. Bioinformatics **25**(14): 1754–1760. doi:10.1093/bioinformatics/btp324.

Purcell, S., Neale, B., Todd-Brown, K., Thomas, L., Ferreira, M.A.R., Bender, D., Maller, J., Sklar, P., de Bakker, P.I.W., Daly, M.J., and Sham, P.C. 2007. PLINK: A tool set for whole-genome association and population-based linkage analyses. Am. J. Hum. Genet. **81**(3): 559–575. doi:10.1086/519795.

Rimmer, A., Phan, H., Mathieson, I., Iqbal, Z., Twigg, S.R.F., WGS500 Consortium, Wilkie, A.O.M., McVean, G., and Lunter, G. 2014. Integrating mapping-, assembly- and haplotype-based approaches for calling variants in clinical sequencing applications. Nat. Genet. **46**(8): 912–918. doi:10.1038/ng.3036.

Smith, J.J., Timoshevskaya, N., Ye, C., Holt, C., Keinath, M.C., Parker, H.J., Cook, M.E., Hess, J.E., Narum, S.R., Lamanna, F., Kaessmann, H., Timoshevskiy, V.A., Waterbury, C.K.M., Saraceno, C., Wiedemann, L.M., Robb, S.M.C., Baker, C., Eichler, E.E., Hockman, D., Sauka-Spengler, T., Yandell, M., Krumlauf, R., Elgar, G., and Amemiya, C.T. 2018. The sea lamprey germline genome provides insights into programmed genome rearrangement and vertebrate evolution. Nat. Genet. **50**(2): 270–277. doi:10.1038/s41588-017-0036-1.

**Section B: Supplemental Figures and Tables**


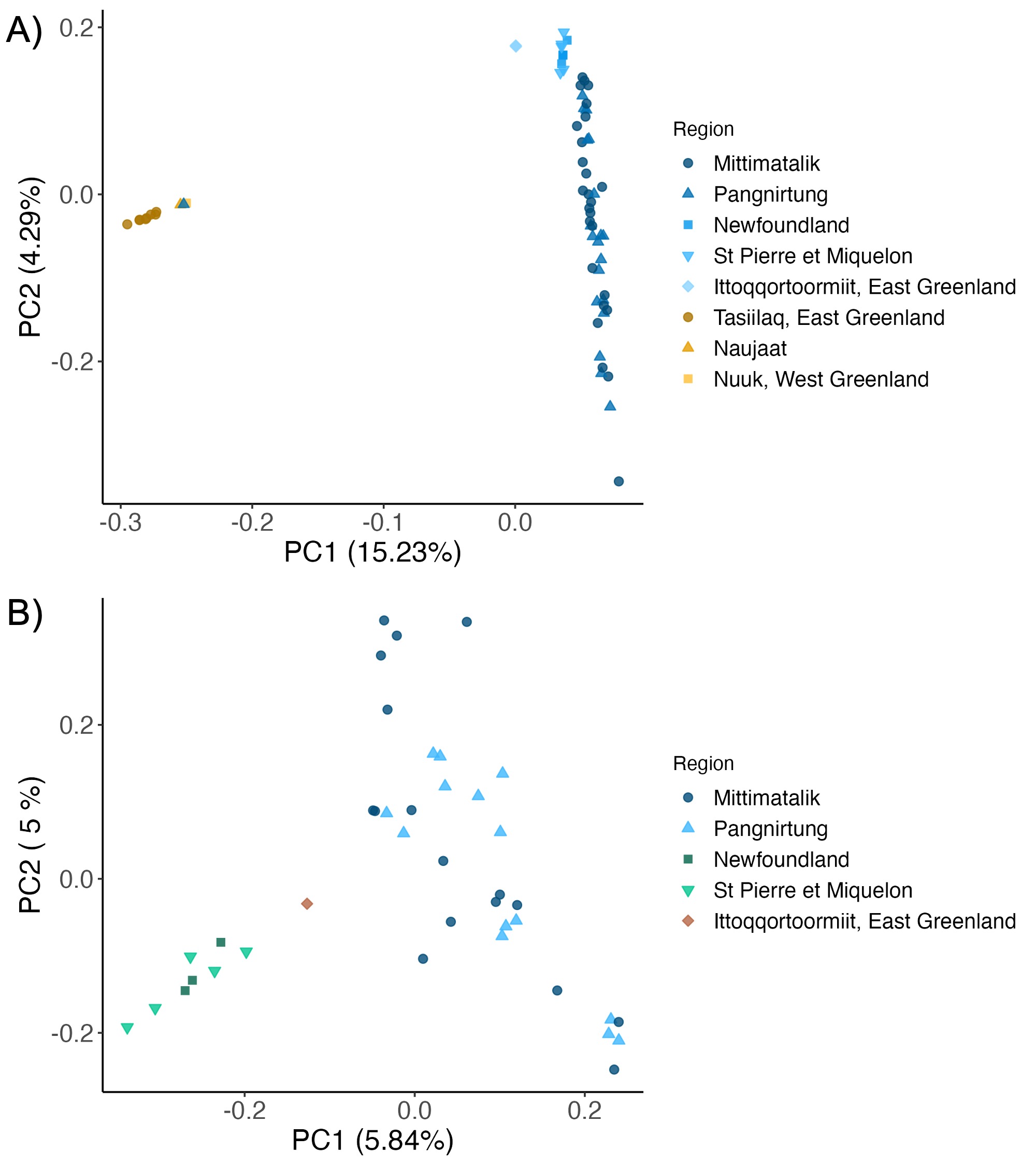


**Supp. Figure 1**. Principal component analysis of whole genome sequence data showing the population structure of killer whales in the eastern Canadian Arctic and Greenland, with (A) no kin removed from either ECAG1 or ECAG2 (n = 67), and (B) including individuals in ECAG1 only (close kin removed; n = 40).


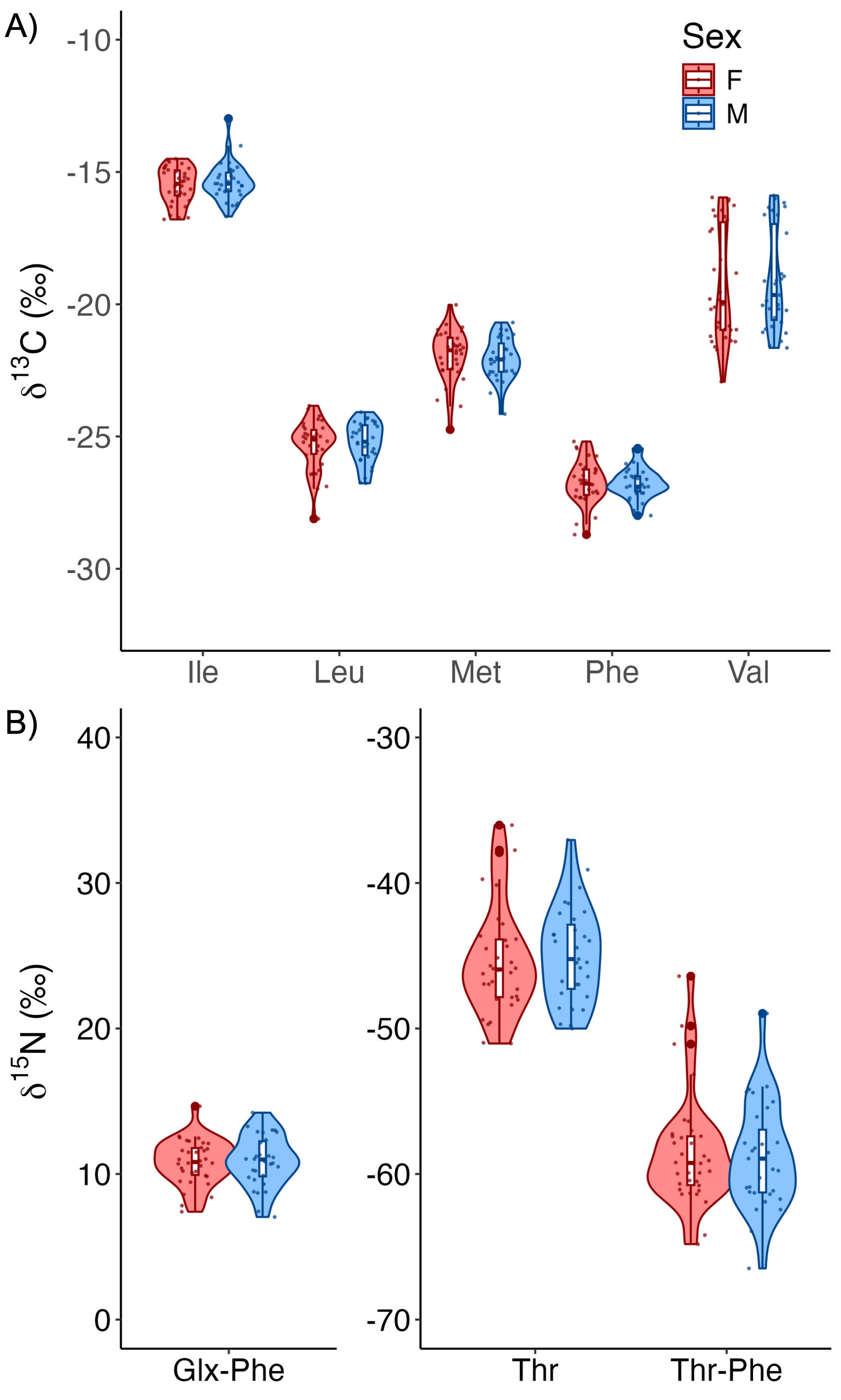


**Supp. Figure 2**. Boxplots showing no difference in (A) δ^13^C_Ile_, δ^13^C_Leu_, δ^13^C_Met_, δ^13^C_Phe_, and δ^13^C_Val_, and (B) δ^15^N_Glx-Phe_, δ^15^N_Thr_, and δ^15^N_Thr-Phe_ values between male (n = 31) and female (n = 34) killer whales sampled in eight locations in the northwest Atlantic between 2012 and 2022.


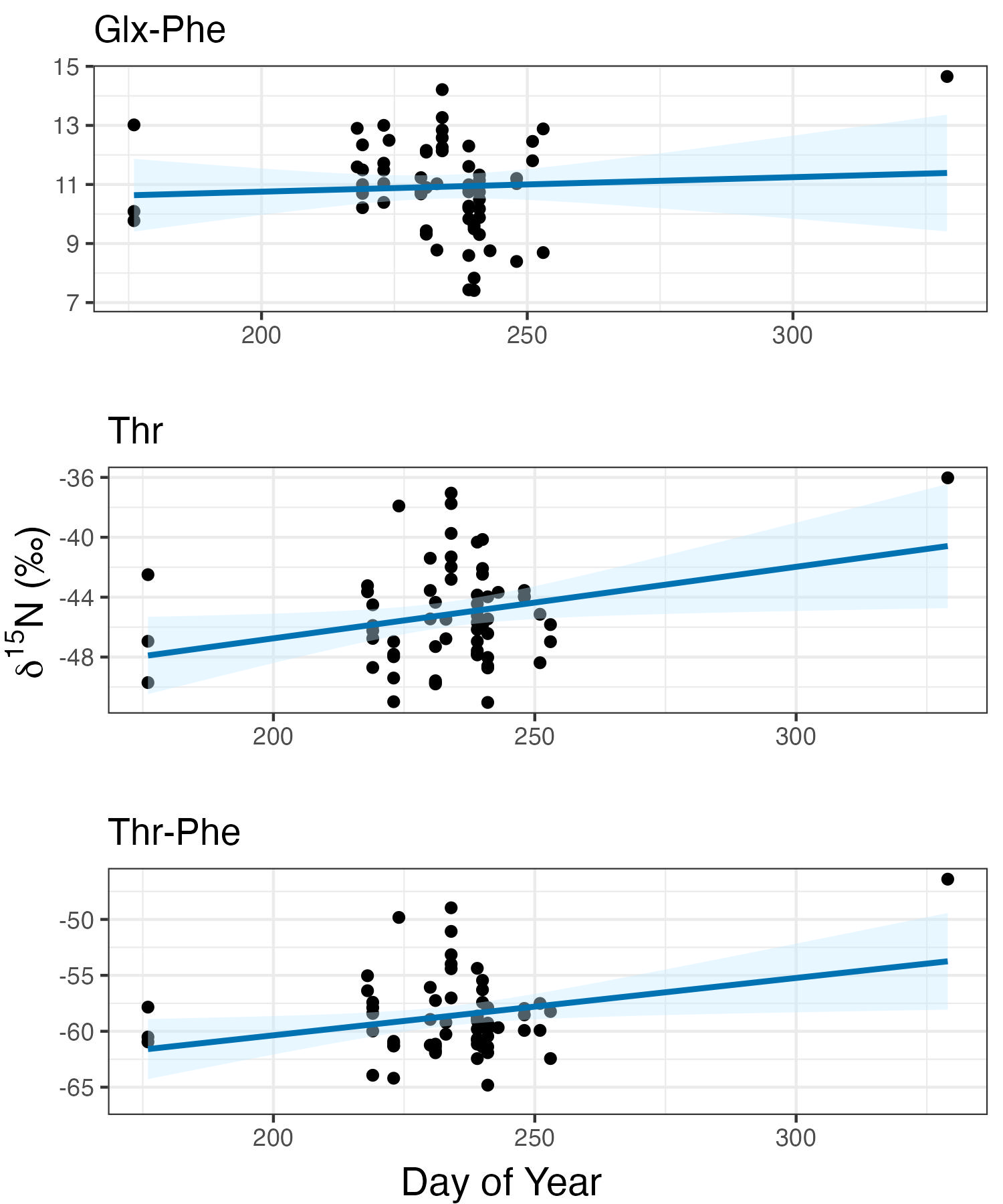


**Supp. Figure 3.** Day of year was not a significant co-variate of genetic group for δ^15^N_Glx-Phe_, δ^15^N_Thr_, or δ^15^N_Thr-Phe_ for 60 killer whales sampled between 2012 and 2022 in the northwest Atlantic. Exact sampling date was not known for five individuals, but they were known to be sampled in July and August (day of year 182-243).


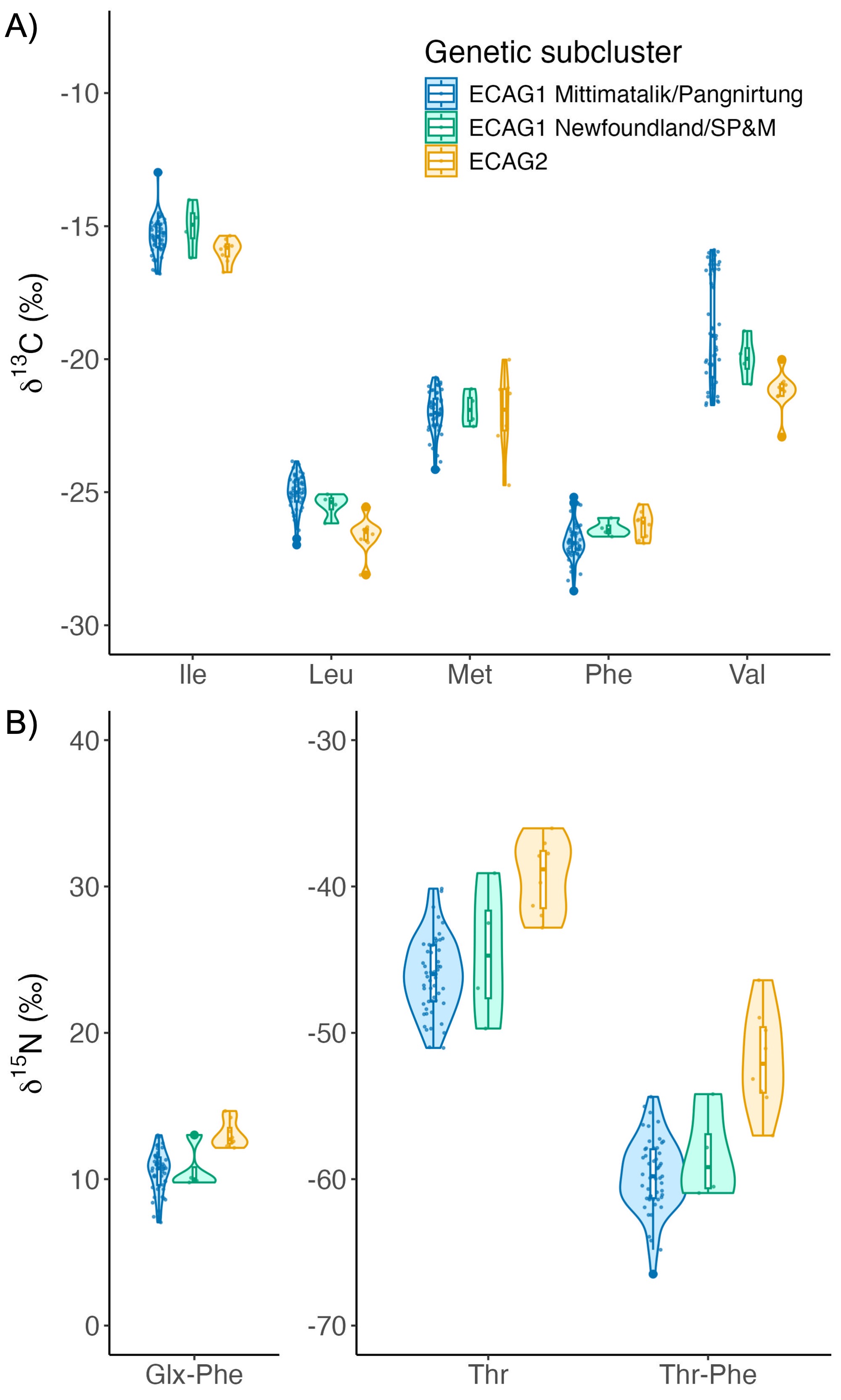


**Supp. Figure 4**. (A) δ^13^C_Ile_, δ^13^C_Leu_, δ^13^C_Met_, δ^13^C_Phe_, and δ^13^C_Val_, and (B) δ^15^N_Glx-Phe_, δ^15^N_Thr_, and δ^15^N_Thr-Phe_ values for genetic population ECAG2 (n = 8) and genetic subclusters in population ECAG1 (sampled in Mittimatalik/Pangnirtung: n = 53, sampled in Newfoundland/St. Pierre et Miquelon: n = 4); Arctic and Atlantic for killer whales skin samples collected between 2012-2022 from eight locations in the northwest Atlantic.


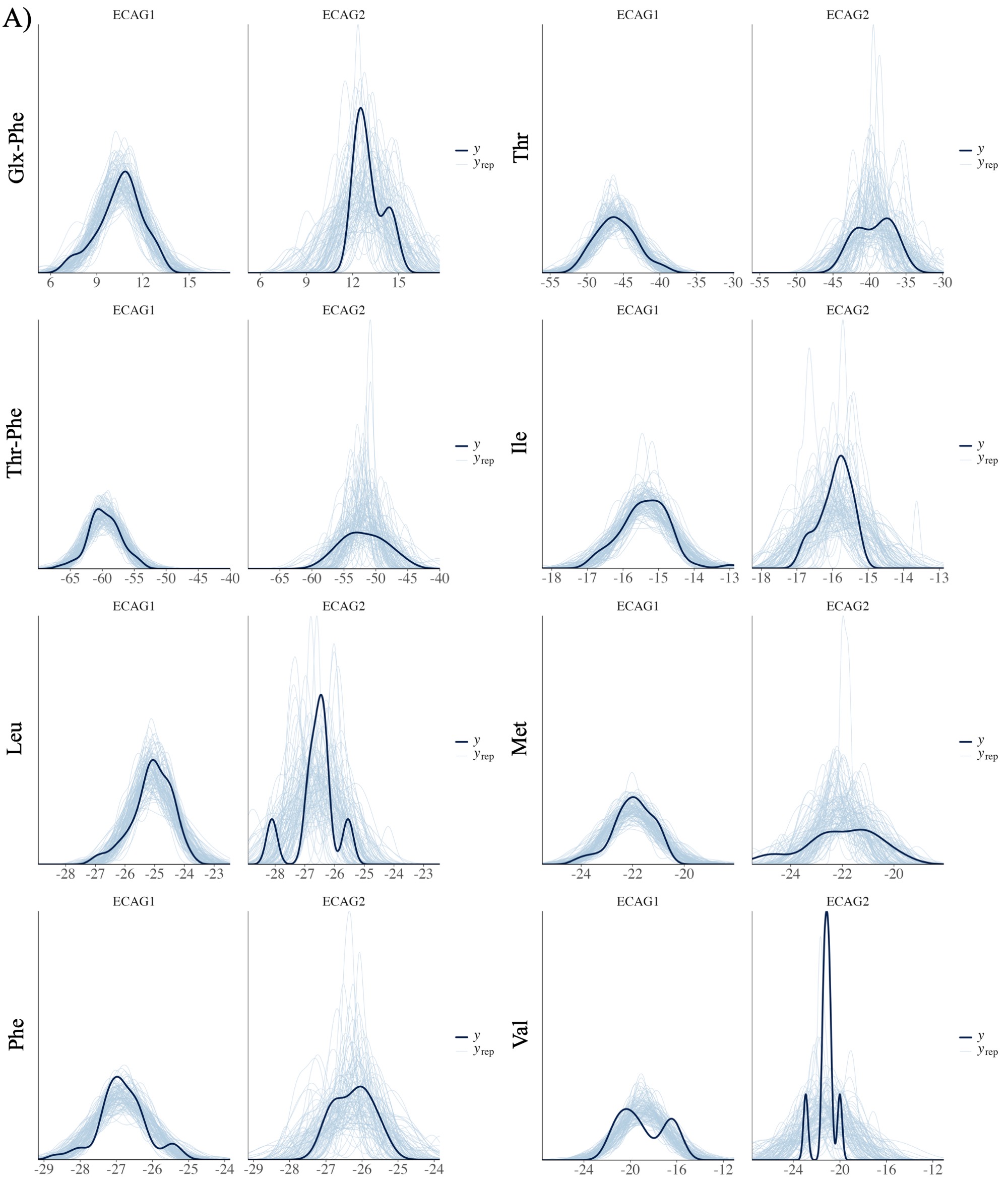


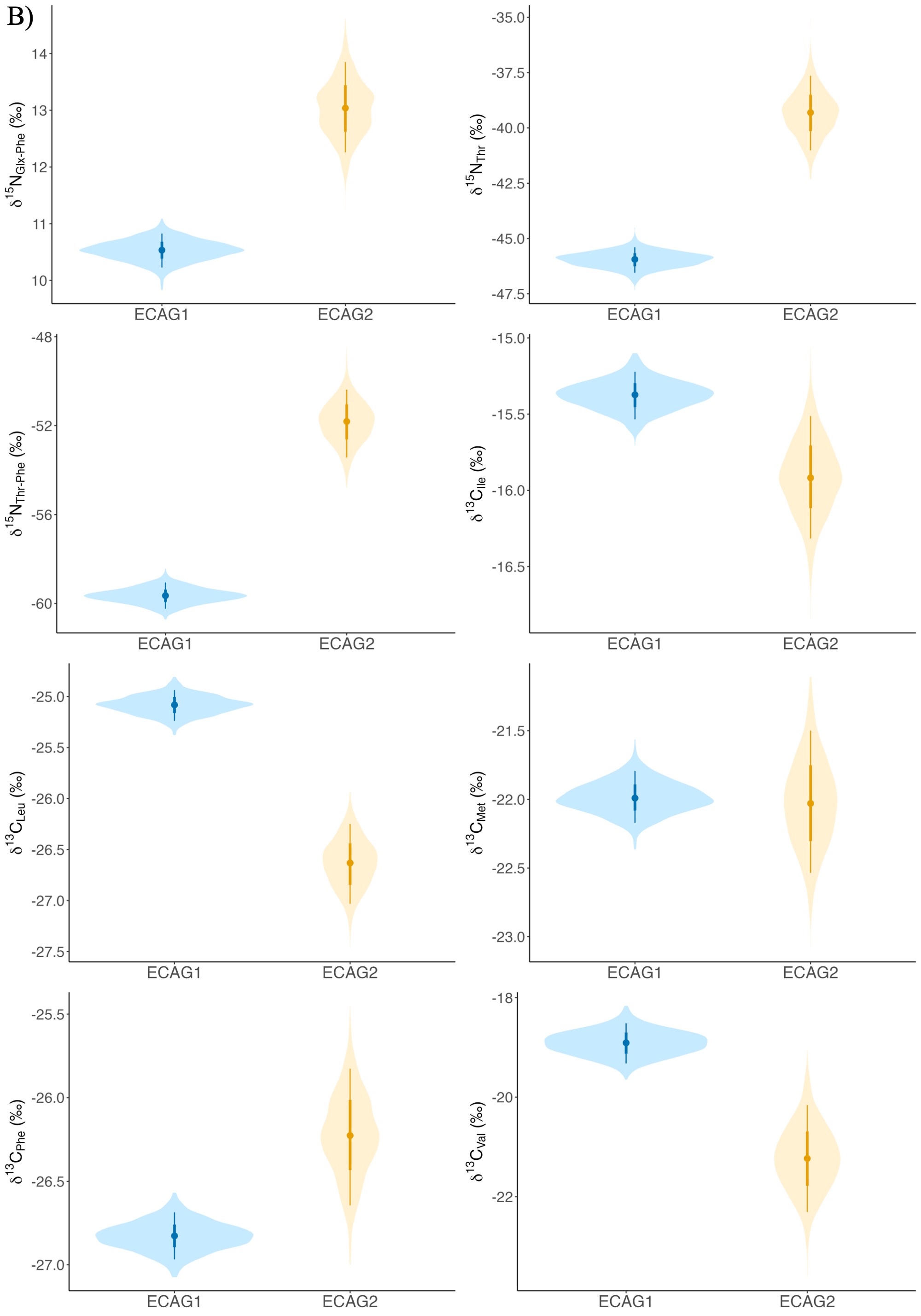


**Supp. Figure 5**. Posterior predictive plots for univariate (δ^15^N_Glx-Phe_, δ^15^N_Thr_, δ^15^N_Thr-Phe_) and multivariate (δ^13^C_Ile_, δ^13^C_Leu_, δ^13^C_Met_, δ^13^C_Phe_, and δ^13^C_Val_) models examining differences between genetic groups ECAG1 and ECAG2. The A) posterior distribution of the mean AA value from the model (thick dark blue line) overlayed on the distribution of the mean AA value of 100 datasets simulated from the model (thin light blue lines) for ECAG1 and ECAG2, and B) the spread of model draws for ECAG1 (blue) and ECAG2 (orange), indicate lower certainty and model predictive ability for ECAG2 posterior estimates than ECAG1.

**Supp. Table 1**. List of killer whale skin samples collected for whole genome sequencing (WGS; n = 90) and compound-specific stable isotope analysis of amino acids (CSIA-AA; n = 81) between 2008 and 2022 from eight locations across the northwest Atlantic: Mittimatalik (Pond Inlet), Naujaat, and Pangnirtung in Nunavut, Canada; Newfoundland and St. Pierre et Miquelon (St. P&M), France on the southern Atlantic coast of Canada; Nuuk in West Greenland; and Ittoqqortoormiit and Tasiilaq in East Greenland. Individuals re-sampled in the same year or in different years are indicated by their sample ID, and were removed from the WGS analysis (WGS duplicate removed; n = 24) and CSIA-AA (CSIA duplicate removed; n = 16). Close kin were also removed from ECAG1 only for the WGS analysis (WGS close kin removed; n = 15). Individuals that were determined from the WGS analysis to be in ECAG2 are bolded.

| **Sample ID** | **Year** | **Location** | **Sex** | **Duplicate ID(s)** | **WGS** | **WGS Duplicate Removed** | **WGS Close Kin Removed** | **CSIA** | **CSIA Duplicate Removed** |
| --- | --- | --- | --- | --- | --- | --- | --- | --- | --- |
| KW-BIOP-08-01 | 2008 | Newfoundland | M |  | x |  |  |  |  |
| **ARRB_xx_1291** | **2009** | **Naujaat** | **F** |  | **x** |  |  |  |  |
| **48335** | **2012** | **Tasiilaq, EG** | **F** |  | **x** |  |  |  |  |
| **48336** | **2012** | **Tasiilaq, EG** | **F** |  | **x** |  |  | **x** |  |
| **48337** | **2012** | **Tasiilaq, EG** | **M** |  | **x** |  |  | **x** |  |
| **48338** | **2012** | **Tasiilaq, EG** | **F** |  | **x** |  |  | **x** |  |
| **48339** | **2012** | **Tasiilaq, EG** | **M** |  | **x** |  |  | **x** |  |
| **48340** | **2012** | **Tasiilaq, EG** | **M** |  | **x** |  |  |  |  |
| **48341** | **2012** | **Tasiilaq, EG** | **M** |  | **x** |  |  | **x** |  |
| **48342** | **2012** | **Tasiilaq, EG** | **F** |  | **x** |  |  | **x** |  |
| **ARPG-2013-01** | **2013** | **Pangnirtung** | **F** |  | **x** |  |  | **x** |  |
| ARPI-2013-01 | 2013 | Mittimatalik | F | KW-2020-PG-21 | x | x |  | x |  |
| ARPI-2013-02 | 2013 | Mittimatalik | M |  | x |  | x |  |  |
| ARPI-2013-03 | 2013 | Mittimatalik | F | ARPI-2013-04, KW-2021-PG-11 | x | x |  | x |  |
| ARPI-2013-04 | 2013 | Mittimatalik | F | ARPI-2013-03, KW-2021-PG-11 | x | x |  | x | x |
| ARPI-2013-05 | 2013 | Mittimatalik | M | KW-2021-PG-08, KW-2021-PG-XX | x | x |  | x |  |
| **Sample ID** | **Year** | **Location** | **Sex** | **Duplicate ID(s)** | **WGS** | **WGS Duplicate Removed** | **WGS Close Kin Removed** | **CSIA** | **CSIA Duplicate Removed** |
| ARPI-2013-06 | 2013 | Mittimatalik | F | KW-2020-PG-06, KW-2020-PG-18 | x | x |  | x |  |
| ARPI-2013-07 | 2013 | Mittimatalik | M | KW-2021-PG-09 | x | x |  | x |  |
| ARPI-2018-01 | 2018 | Mittimatalik | F |  | x |  |  | x |  |
| ARPI-2018-02 | 2018 | Mittimatalik | M | ARPI-2018-17, KW-2021-PG-06 | x | x |  | x | x |
| ARPI-2018-03 | 2018 | Mittimatalik | F |  |  |  |  | x |  |
| ARPI-2018-05 | 2018 | Mittimatalik | M |  |  |  |  | x |  |
| ARPI-2018-06 | 2018 | Mittimatalik | F |  |  |  |  | x |  |
| ARPI-2018-07 | 2018 | Mittimatalik | F | KW-2020-PI-12 | x | x |  |  |  |
| ARPI-2018-10 | 2018 | Mittimatalik | F | ARPI-2018-14 | x | x |  | x |  |
| ARPI-2018-11 | 2018 | Mittimatalik | F | KW-2020-PI-08 | x |  | x |  |  |
| ARPI-2018-13 | 2018 | Mittimatalik | F | KW-2022-PI-01 | x |  | x | x |  |
| ARPI-2018-14 | 2018 | Mittimatalik | F | ARPI-2018-10 | x |  |  |  |  |
| ARPI-2018-15 | 2018 | Mittimatalik | M | ARPI-2018-16 | x |  |  | x |  |
| ARPI-2018-16 | 2018 | Mittimatalik | M | ARPI-2018-15 | x | x |  |  |  |
| ARPI-2018-17 | 2018 | Mittimatalik | M | ARPI-2018-02, KW-2021-PG-06 | x | x |  |  |  |
| ARPI-2018-18 | 2018 | Mittimatalik | M |  | x |  | x | x |  |
| ARPI-2019-01 | 2019 | Mittimatalik | M |  | x |  |  | x |  |
| ARPI-2019-02 | 2019 | Mittimatalik | M |  | x |  |  | x |  |
| ARPI-2019-03 | 2019 | Mittimatalik | M | ARPI-2019-05 | x | x |  | x |  |
| ARPI-2019-04 | 2019 | Mittimatalik | F |  | x |  |  | x |  |
| ARPI-2019-05 | 2019 | Mittimatalik | M | ARPI-2019-03 | x |  |  |  |  |
| ARPI-2019-06 | 2019 | Mittimatalik | F |  | x |  |  | x |  |
| ARPI-2019-07 | 2019 | Mittimatalik | M |  | x |  |  | x |  |
| ARPI-2019-08 | 2019 | Mittimatalik | M |  | x |  |  | x |  |
| ARPI-2019-09 | 2019 | Mittimatalik | F |  | x |  |  | x |  |
| ARPI-2019-10 | 2019 | Mittimatalik | M |  | x |  | x | x |  |
| ARPI-2019-11 | 2019 | Mittimatalik | F |  | x |  | x | x |  |
| KW-BIOP-17-01 | 2019 | Newfoundland | M |  | x |  |  |  |  |
| **Sample ID** | **Year** | **Location** | **Sex** | **Duplicate ID(s)** | **WGS** | **WGS Duplicate Removed** | **WGS Close Kin Removed** | **CSIA** | **CSIA Duplicate Removed** |
| KW-2019-01 | 2019 | St. P&M, FR | M |  | x |  |  |  |  |
| KW-2019-02 | 2019 | St. P&M, FR | M |  | x |  |  |  |  |
| KW-2020-PG-01 | 2020 | Pangnirtung | M | KW-2020-PG-12, KW-2021-PG-03 | x | x |  | x | x |
| KW-2020-PG-02 | 2020 | Pangnirtung | F |  | x |  |  | x |  |
| KW-2020-PG-03 | 2020 | Pangnirtung | F | KW-2020-PG-07, KW-2021-PG-05 | x | x |  | x | x |
| KW-2020-PG-04 | 2020 | Pangnirtung | M | KW-2020-PG-20 | x | x |  | x |  |
| KW-2020-PG-05 | 2020 | Pangnirtung | M |  | x |  |  | x |  |
| KW-2020-PG-06 | 2020 | Pangnirtung | F | ARPI-2013-06, KW-2020-PG-18 | x | x |  | x | x |
| KW-2020-PG-07 | 2020 | Pangnirtung | F | KW-2020-PG-03, KW-2021-PG-05 | x | x |  | x | x |
| KW-2020-PG-08 | 2020 | Pangnirtung | F |  |  |  |  | x |  |
| KW-2020-PG-09 | 2020 | Pangnirtung | F | KW-2020-PG-19 | x |  |  | x |  |
| KW-2020-PG-10 | 2020 | Pangnirtung | M |  |  |  |  | x |  |
| KW-2020-PG-11 | 2020 | Pangnirtung | F |  | x |  | x | x |  |
| KW-2020-PG-12 | 2020 | Pangnirtung | M | KW-2020-PG-01, KW-2021-PG-03 | x | x |  | x | x |
| KW-2020-PG-13 | 2020 | Pangnirtung | M |  |  |  |  | x |  |
| KW-2020-PG-14 | 2020 | Pangnirtung | M |  |  |  |  | x |  |
| KW-2020-PG-15 | 2020 | Pangnirtung | F |  | x |  | x | x |  |
| KW-2020-PG-16 | 2020 | Pangnirtung | F |  | x |  | x | x |  |
| KW-2020-PG-17 | 2020 | Pangnirtung | M | KW-2020-PG-23 | x |  |  | x |  |
| KW-2020-PG-18 | 2020 | Pangnirtung | F | ARPI-2013-06, KW-2020-PG-06 | x |  |  | x | x |
| KW-2020-PG-19 | 2020 | Pangnirtung | F | KW-2020-PG-09 | x | x |  | x | x |
| KW-2020-PG-20 | 2020 | Pangnirtung | M | KW-2020-PG-04 | x |  |  | x | x |
| KW-2020-PG-21 | 2020 | Pangnirtung | F | ARPI-2013-01 | x |  | x | x | x |
| KW-2020-PG-22 | 2020 | Pangnirtung | F |  | x |  | x | x |  |
| KW-2020-PG-23 | 2020 | Pangnirtung | M | KW-2020-PG-17 | x | x |  | x | x |
| KW-2020-PG-24 | 2020 | Pangnirtung | M |  | x |  |  | x |  |
| KW-2020-PI-02 | 2020 | Mittimatalik | F |  | x |  |  | x |  |
| KW-2020-PI-03 | 2020 | Mittimatalik | M |  | x |  |  | x |  |
| **Sample ID** | **Year** | **Location** | **Sex** | **Duplicate ID(s)** | **WGS** | **WGS Duplicate Removed** | **WGS Close Kin Removed** | **CSIA** | **CSIA Duplicate Removed** |
| KW-2020-PI-05 | 2020 | Mittimatalik | F | KW-2020-PI-06 | x | x |  | x |  |
| KW-2020-PI-06 | 2020 | Mittimatalik | F | KW-2020-PI-05 | x |  | x | x | x |
| KW-2020-PI-07 | 2020 | Mittimatalik | F |  | x |  |  | x |  |
| KW-2020-PI-08 | 2020 | Mittimatalik | F | ARPI-2018-11 | x | x |  | x |  |
| KW-2020-PI-10 | 2020 | Mittimatalik | M |  | x |  |  | x |  |
| KW-2020-PI-12 | 2020 | Mittimatalik | F | ARPI-2018-07 | x |  | x | x |  |
| KW-2020-PI-13 | 2020 | Mittimatalik | M |  | x |  |  | x |  |
| KW-2020-PI-14 | 2020 | Mittimatalik | M |  | x |  |  | x |  |
| KW-2020-PI-15 | 2020 | Mittimatalik | F |  | x |  | x | x |  |
| GRNL-KW-2021-01 | 2021 | Ittoqqortoormiit, EG | F |  | x |  | x | x |  |
| **GRNL-KW-2021-02** | **2021** | **Nuuk, WG** | **F** |  | **x** |  |  | **x** |  |
| GRNL-KW-2021-03 | 2021 | Ittoqqortoormiit, EG | M |  | x |  |  | x |  |
| KW-2021-PG-03 | 2021 | Pangnirtung | M | KW-2020-PG-01, KW-2020-PG-12 | x |  |  | x |  |
| KW-2021-PG-04 | 2021 | Pangnirtung | F |  | x |  |  | x |  |
| KW-2021-PG-05 | 2021 | Pangnirtung | F | KW-2020-PG-03, KW-2020-PG-07 | x |  |  | x |  |
| KW-2021-PG-06 | 2021 | Pangnirtung | M | ARPI-2018-02, ARPI-2018-17 | x |  |  | x |  |
| KW-2021-PG-08 | 2021 | Pangnirtung | M | ARPI-2013-05, KW-2021-PG-XX | x | x |  | x | x |
| KW-2021-PG-09 | 2021 | Pangnirtung | M | ARPI-2013-07, ARPI-2013-03 | x |  |  |  |  |
| KW-2021-PG-11 | 2021 | Pangnirtung | F | ARPI-2013-03, ARPI-2013-04 | x |  |  |  |  |
| KW-2021-PG-XX | 2021 | Pangnirtung | M | ARPI-2013-05, KW-2021-PG-08 | x |  |  | x | x |
| Or21-1 | 2021 | St. P&M, FR | F |  | x |  |  | x |  |
| Or21-2 | 2021 | St. P&M, FR | M |  | x |  |  | x |  |
| Or21-3 | 2021 | St. P&M, FR | M |  | x |  |  | x |  |
| KW-2022-PI-01 | 2022 | Mittimatalik | F | ARPI-2018-13 | x | x |  | x | x |
| KW-Nfld-22-25 | 2022 | Newfoundland | M |  | x |  |  |  |  |

**Supp. Table 2**. Raw compound-specific δ^15^N and δ^13^C stable isotope ratios from 81 killer whale skin samples collected from the northwest Atlantic between 2012-2022. δ^15^N_Glx-Phe_, δ^15^N_Thr_, and δ^15^N_Thr-Phe_ differ between consumer tissues and their prey and thus are used to infer trophic level, while δ^15^N_Phe_ and ^15^N_Lys_ are “source” AAs used to infer distribution. Ile, Leu, Met, Phe, and Val are considered “essential” AAs since they cannot be produced by consumers, and thus their δ^13^C values reflect the source carbon and are used to infer distribution.

|  | δ^15^N_AA_ | | | | | δ^13^C_AA_ | | | | |
| --- | --- | --- | --- | --- | --- | --- | --- | --- | --- | --- |
| Sample ID | **Glx-Phe** | **Thr** | **Thr-Phe** | **Phe** | **Lys** | **Ile** | **Leu** | **Met** | **Phe** | **Val** |
| 48336 | 12.58 | −39.74 | −53.15 | 13.41 | 8.23 | −16.08 | −26.41 | −21.14 | −26.22 | −21.38 |
| 48337 | 14.21 | −41.98 | −54.41 | 12.43 | 8.70 | −15.49 | −26.58 | −22.88 | −26.92 | −20.84 |
| 48338 | 12.26 | −37.75 | −51.07 | 13.32 | 8.30 | −15.76 | −26.41 | −21.29 | −25.74 | −21.21 |
| 48339 | 12.84 | −37.05 | −48.96 | 11.91 | 7.86 | −15.74 | −26.31 | −22.62 | −26.03 | −21.06 |
| 48341 | 13.27 | −41.31 | −53.99 | 12.68 | 8.07 | −15.86 | −26.77 | −22.51 | −26.82 | −21.40 |
| 48342 | 12.14 | −42.81 | −57.02 | 14.21 | 9.48 | −15.36 | −25.55 | −20.02 | −25.46 | −20.01 |
| ARPG-2013-01 | 12.50 | −37.91 | −49.82 | 11.92 | 6.49 | −16.31 | −26.89 | −21.08 | −26.06 | −20.97 |
| ARPI-2013-01 | 10.39 | −47.98 | −61.32 | 13.34 | 6.69 | −14.62 | −24.23 | −21.16 | −25.40 | −18.69 |
| ARPI-2013-03 | 11.72 | −49.40 | −61.07 | 11.67 | 6.44 | −14.50 | −23.84 | −21.64 | −25.19 | −18.31 |
| ARPI-2013-04 | 11.91 | −49.71 | −62.05 | 12.34 | 6.27 | −15.08 | −24.08 | −21.94 | −25.49 | −18.42 |
| ARPI-2013-05 | 11.03 | −46.97 | −61.30 | 14.33 | 6.82 | −14.90 | −24.44 | −20.94 | −25.45 | −19.12 |
| ARPI-2013-06 | 11.48 | −50.98 | −64.20 | 13.22 | 7.07 | −15.26 | −24.39 | −21.75 | −25.71 | −18.82 |
| ARPI-2013-07 | 13.00 | −47.82 | −60.90 | 13.08 | 8.10 | −14.92 | −24.63 | −21.14 | −25.48 | −18.85 |
| ARPI-2018-01 | 10.75 | −47.85 | −60.79 | 12.94 | 7.08 | −14.82 | −25.12 | −22.24 | −26.28 | −20.96 |
| ARPI-2018-02 | 11.79 | −47.70 | −60.69 | 12.99 | 7.87 | −14.78 | −25.00 | −22.90 | −26.56 | −20.34 |
| ARPI-2018-03 | 7.43 | −45.23 | −62.44 | 17.21 | 8.26 | −15.52 | −24.76 | −20.69 | −26.54 | −20.23 |
| ARPI-2018-05 | 10.20 | −45.69 | −60.68 | 14.99 | 7.65 | −14.98 | −25.01 | −21.02 | −26.81 | −20.96 |
| ARPI-2018-06 | 11.61 | −47.79 | −59.81 | 12.02 | 7.40 | −14.74 | −24.35 | −20.87 | −26.24 | −19.55 |
| ARPI-2018-10 | 11.80 | −45.14 | −57.51 | 12.37 | 7.26 | −15.03 | −24.62 | −21.59 | −26.38 | −20.19 |
| ARPI-2018-13 | 12.46 | −48.38 | −59.92 | 11.54 | 7.27 | −14.92 | −24.68 | −23.22 | −26.48 | −20.69 |
| ARPI-2018-15 | 8.69 | −45.83 | −62.44 | 16.61 | 8.85 | −15.36 | −25.04 | −20.98 | −26.50 | −20.04 |
| ARPI-2018-18 | 12.88 | −46.97 | −58.23 | 11.26 | 7.07 | −14.66 | −24.62 | −22.68 | −27.14 | −19.10 |
| ARPI-2019-01 | 10.74 | −43.55 | −58.94 | 15.39 | 7.41 | −15.24 | −25.77 | −21.71 | −27.54 | −20.51 |
|  |  |  | **δ^15^N_AA_** |  |  |  |  | **δ^13^C_AA_** |  |  |
| Sample ID | **Glx-Phe** | **Thr** | **Thr-Phe** | **Phe** | **Lys** | **Ile** | **Leu** | **Met** | **Phe** | **Val** |
| ARPI-2019-02 | 10.68 | −45.45 | −61.24 | 15.79 | 8.48 | −14.65 | −24.85 | −21.89 | −26.94 | −19.23 |
| ARPI-2019-03 | 11.23 | −41.40 | −56.07 | 14.67 | 8.82 | −15.52 | −25.90 | −22.08 | −27.42 | −20.02 |
| ARPI-2019-04 | 9.32 | −47.30 | −61.40 | 14.10 | 5.41 | −16.73 | −26.28 | −23.63 | −28.08 | −20.82 |
| ARPI-2019-06 | 11.02 | −46.78 | −59.21 | 12.44 | 5.92 | −16.64 | −26.98 | −23.86 | −28.71 | −21.73 |
| ARPI-2019-07 | 8.78 | −45.48 | −60.27 | 14.79 | 6.83 | −16.27 | −26.75 | −22.56 | −27.99 | −21.57 |
| ARPI-2019-08 | 11.14 | −48.60 | −61.39 | 12.79 | 5.08 | −15.66 | −25.87 | −22.66 | −27.80 | −20.68 |
| ARPI-2019-09 | 9.88 | −51.03 | −64.82 | 13.79 | 5.66 | −15.19 | −26.43 | −22.83 | −28.32 | −21.41 |
| ARPI-2019-10 | 11.32 | −48.73 | −61.91 | 13.19 | 6.46 | −15.44 | −25.29 | −21.24 | −26.91 | −21.09 |
| ARPI-2019-11 | 10.75 | −48.03 | −60.45 | 12.42 | 5.12 | −15.53 | −25.70 | −22.56 | −27.32 | −20.87 |
| KW-2020-PG-01 | 12.63 | −47.19 | −59.45 | 12.26 | 7.55 | −15.08 | −24.48 | −22.00 | −26.47 | −16.88 |
| KW-2020-PG-02 | 11.60 | −43.64 | −56.38 | 12.74 | 8.67 | −15.06 | −23.96 | −21.26 | −26.66 | −16.44 |
| KW-2020-PG-03 | 12.18 | −47.33 | −59.05 | 11.72 | 7.71 | −15.64 | −24.41 | −22.53 | −27.00 | −16.91 |
| KW-2020-PG-04 | 12.90 | −43.23 | −55.04 | 11.80 | 8.24 | −15.71 | −24.08 | −22.09 | −26.92 | −17.31 |
| KW-2020-PG-05 | 12.34 | −46.75 | −58.41 | 11.65 | 7.27 | −15.39 | −24.53 | −21.28 | −26.34 | −16.34 |
| KW-2020-PG-06 | 10.88 | −48.45 | −61.54 | 13.09 | 6.89 | −15.19 | −24.19 | −20.84 | −26.27 | −16.09 |
| KW-2020-PG-07 | 12.52 | −47.05 | −58.53 | 11.49 | 7.91 | −16.20 | −25.09 | −22.17 | −27.07 | −17.07 |
| KW-2020-PG-08 | 11.00 | −44.51 | −57.41 | 12.90 | 7.46 | −15.88 | −24.50 | −22.02 | −26.82 | −17.15 |
| KW-2020-PG-09 | 10.70 | −46.23 | −59.98 | 13.74 | 7.80 | −15.79 | −24.71 | −21.49 | −27.31 | −16.66 |
| KW-2020-PG-10 | 10.21 | −48.70 | −63.93 | 15.24 | 9.29 | −14.95 | −24.44 | −22.94 | −26.24 | −16.16 |
| KW-2020-PG-11 | 11.49 | −45.89 | −57.89 | 12.00 | 7.40 | −16.31 | −25.35 | −22.26 | −27.27 | −17.24 |
| KW-2020-PG-12 | 12.48 | −46.79 | −59.02 | 12.23 | 7.47 | −16.27 | −24.91 | −23.02 | −27.44 | −17.01 |
| KW-2020-PG-13 | 10.86 | −44.44 | −58.94 | 14.50 | 8.24 | −16.18 | −25.23 | −21.82 | −27.14 | −16.62 |
| KW-2020-PG-14 | 10.99 | −47.57 | −61.16 | 13.58 | 7.73 | −16.27 | −25.20 | −22.17 | −26.91 | −16.45 |
| KW-2020-PG-15 | 12.30 | −46.96 | −58.77 | 11.81 | 7.28 | −15.84 | −24.94 | −22.45 | −26.79 | −16.44 |
| KW-2020-PG-16 | 9.84 | −46.16 | −58.64 | 12.48 | 8.06 | −16.79 | −25.36 | −22.49 | −27.07 | −16.81 |
| KW-2020-PG-17 | 10.27 | −40.32 | −54.37 | 14.05 | 8.65 | −16.68 | −25.65 | −22.09 | −27.16 | −16.61 |
| KW-2020-PG-18 | 8.99 | −47.07 | −61.75 | 14.68 | 7.28 | −16.36 | −25.17 | −22.15 | −27.20 | −16.09 |
| KW-2020-PG-19 | 9.41 | −45.36 | −59.77 | 14.41 | 7.22 | −16.35 | −25.13 | −22.16 | −27.31 | −15.88 |
| KW-2020-PG-20 | 11.16 | −45.80 | −58.52 | 12.72 | 7.45 | −16.36 | −25.11 | −22.48 | −27.09 | −15.93 |
| KW-2020-PG-21 | 9.24 | −45.58 | −59.63 | 14.05 | 7.19 | −15.91 | −24.95 | −21.88 | −26.75 | −15.84 |
|  |  |  | **δ^15^N_AA_** |  |  |  |  | **δ^13^C_AA_** |  |  |
| Sample ID | **Glx-Phe** | **Thr** | **Thr-Phe** | **Phe** | **Lys** | **Ile** | **Leu** | **Met** | **Phe** | **Val** |
| KW-2020-PG-22 | 8.60 | −43.86 | −59.08 | 15.22 | 7.97 | −15.89 | −25.18 | −21.85 | −26.78 | −16.26 |
| KW-2020-PG-23 | 10.41 | −41.26 | −55.56 | 14.30 | 6.61 | −15.95 | −25.28 | −22.48 | −27.35 | −16.35 |
| KW-2020-PG-24 | 8.76 | −43.68 | −59.67 | 16.00 | 8.47 | −15.67 | −24.52 | −21.17 | −26.62 | −16.00 |
| KW-2020-PI-02 | 9.43 | −44.34 | −57.25 | 12.91 | 5.93 | −15.75 | −24.89 | −21.87 | −27.01 | −16.09 |
| KW-2020-PI-03 | 9.60 | −42.08 | −55.45 | 13.37 | 5.86 | −15.14 | −24.33 | −22.02 | −26.90 | −15.89 |
| KW-2020-PI-05 | 7.41 | −40.15 | −56.29 | 16.14 | 6.41 | −15.54 | −25.49 | −21.53 | −27.37 | −16.68 |
| KW-2020-PI-06 | 7.85 | −40.46 | −56.86 | 16.40 | 9.39 | −15.33 | −25.24 | −21.44 | −27.14 | −16.66 |
| KW-2020-PI-07 | 9.50 | −42.47 | −57.42 | 14.95 | 6.92 | −14.86 | −25.07 | −20.96 | −27.03 | −15.96 |
| KW-2020-PI-08 | 7.83 | −45.85 | −61.39 | 15.54 | 6.04 | −15.24 | −25.01 | −20.76 | −27.12 | −16.02 |
| KW-2020-PI-10 | 9.30 | −43.97 | −57.88 | 13.91 | 5.73 | −15.44 | −24.40 | −21.68 | −27.33 | −16.30 |
| KW-2020-PI-12 | 10.17 | −45.45 | −59.27 | 13.82 | 8.07 | −15.65 | −25.14 | −22.50 | −27.24 | −21.41 |
| KW-2020-PI-13 | 11.20 | −44.00 | −58.53 | 14.53 | 8.90 | −15.83 | −24.30 | −24.15 | −27.02 | −19.04 |
| KW-2020-PI-14 | 11.03 | −43.55 | −57.96 | 14.41 | 8.96 | −15.71 | −25.02 | −22.04 | −26.87 | −19.87 |
| KW-2020-PI-15 | 8.39 | −43.93 | −59.93 | 15.99 | 9.15 | −16.11 | −25.22 | −22.09 | −27.32 | −19.83 |
| GRNL-KW-2021-01 | 10.56 | −45.98 | −57.87 | 11.89 | 5.85 | −15.34 | −26.05 | −21.61 | −26.16 | −21.61 |
| GRNL-KW-2021-02 | 14.66 | −36.03 | −46.40 | 10.37 | 6.31 | −16.73 | −28.11 | −24.74 | −26.65 | −22.93 |
| GRNL-KW-2021-03 | 7.05 | −50.00 | −66.48 | 16.48 | 6.30 | −12.98 | −25.39 | −23.36 | −26.93 | −20.18 |
| KW-2021-PG-03 | 12.15 | −49.79 | −61.73 | 11.94 | 4.98 | −14.82 | −24.88 | −22.55 | −26.59 | −19.65 |
| KW-2021-PG-04 | 12.10 | −49.71 | −61.15 | 11.44 | 4.06 | −14.86 | −24.90 | −21.73 | −26.51 | −20.11 |
| KW-2021-PG-05 | 10.90 | −49.58 | −61.92 | 12.34 | 6.54 | −14.56 | −25.00 | −22.46 | −27.15 | −21.26 |
| KW-2021-PG-06 | 10.50 | −46.43 | −59.77 | 13.34 | 5.92 | −15.10 | −25.58 | −22.09 | −26.55 | −21.65 |
| KW-2021-PG-08 | 9.64 | −45.93 | −61.16 | 15.23 | 6.16 | −15.21 | −25.61 | −21.49 | −26.48 | −21.16 |
| KW-2021-PG-XX | 9.58 | −46.73 | −61.54 | 14.81 | 5.97 | −14.87 | −25.58 | −21.84 | −26.52 | −20.60 |
| OR21-1 | 10.09 | −46.94 | −60.52 | 13.57 | 5.06 | −14.68 | −25.08 | −21.57 | −26.6 | −19.80 |
| OR21-2 | 9.77 | −42.50 | −57.84 | 15.35 | 8.25 | −15.21 | −26.17 | −22.25 | −26.50 | −20.94 |
| OR21-3 | 13.02 | −49.70 | −60.95 | 11.25 | 6.54 | −14.01 | −25.47 | −22.53 | −25.97 | −20.17 |
| KW-2022-PI-01 | 10.61 | −45.01 | −60.81 | 15.80 | 7.68 | −14.72 | −26.45 | −23.09 | −26.54 | −20.40 |
| KW-Nfld-22-25 | 9.89 | −39.09 | −54.19 | 15.10 | 7.20 | −16.19 | −25.28 | −21.12 | −26.35 | −18.94 |

**Supp. Table 3.** Results from multivariate (δ^13^C of essential amino acids Ile, Leu, Met, Phe, and Val) and univariate (δ^15^N of trophic and source amino acids Glx-Phe, Thr, Thr-Phe) models examining differences in compound-specific stable isotope ratios between genetic populations ECAG1 and ECAG2 and sex, measured in 65 killer whale skin samples collected between 2012 and 2022 from eight locations across the northwest Atlantic. For each AA or paired AA examined, ECAG2 was interpreted to significantly differ from ECAG1, and males to significantly differ from females, when the 95% Bayesian credible interval (L = lower limit, U = upper limit) for ECAG2 or males did not overlap with zero. AAs that differed are in italics and indicated with an asterisk.

| **Model** | **Coefficients** | **Estimate** | **Est. Error** | **L-95% C.I.** | **U-95% C.I.** |  |
| --- | --- | --- | --- | --- | --- | --- |
| δ^13^C AA_ESS_ ~ genetic population + sex | Ile Intercept | −15.43 | 0.13 | −15.68 | −15.18 |  |
|  | Leu Intercept | −25.09 | 0.13 | −25.34 | −24.85 |  |
|  | Met Intercept | −21.92 | 0.16 | −22.24 | −21.59 |  |
|  | Phe Intercept | −26.83 | 0.12 | −27.08 | −26.59 |  |
|  | Val Intercept | −18.94 | 0.35 | −19.63 | −18.25 |  |
|  | Ile Genetic Population ECAG2 | −0.53 | 0.27 | −1.05 | 0.00 |  |
|  | Ile Sex Male | 0.11 | 0.18 | −0.23 | 0.46 |  |
|  | *Leu Genetic Population ECAG2* | −*1.54* | *0.26* | −*2.06* | −*1.03* | * |
|  | Leu Sex Male | 0.01 | 0.17 | −0.33 | 0.35 |  |
|  | Met Genetic Population ECAG2 | −0.06 | 0.34 | −0.74 | 0.62 |  |
|  | Met Sex Male | −0.14 | 0.23 | −0.59 | 0.31 |  |
|  | *Phe Genetic Population ECAG2* | *0.59* | *0.26* | *0.08* | *1.10* | *** |
|  | Phe Sex Male | 0.01 | 0.17 | −0.33 | 0.34 |  |
|  | *Val Genetic Population ECAG2* | −*2.32* | *0.74* | −*3.78* | −*0.86* | *** |
|  | Val Sex Male | 0.06 | 0.49 | −0.90 | 1.03 |  |
| δ^15^N_Glx-Phe_ ~ genetic population + sex | Intercept | 10.43 | 0.26 | 9.92 | 10.94 |  |
|  | *Genetic Population ECAG2* | *2.55* | *0.54* | *1.49* | *3.62* | * |
|  | Sex Male | 0.19 | 0.35 | −0.50 | 0.89 |  |
| δ^15^N_Thr_ ~ genetic population + sex | Intercept | −46.36 | 0.50 | −7.33 | −45.39 |  |
|  | *Genetic Population ECAG2* | *6.72* | *1.04* | *4.68* | *8.78* | * |
|  | Sex Male | 0.79 | 0.68 | −0.56 | 2.14 |  |
| δ^15^N_Thr-Phe_ ~ genetic population + sex | Intercept | −59.68 | 0.48 | −60.62 | −58.73 |  |
|  | *Genetic Population ECAG2* | *7.80* | *1.01* | *5.81* | *9.80* | * |
|  | Sex Male | 0.05 | 0.67 | −1.27 | 1.36 |  |

**Supp. Table 4.** Results from multivariate (δ^13^C of essential amino acids Ile, Leu, Met, Phe, and Val) and univariate (δ^15^N of trophic and source amino acids Glx-Phe, Thr, Thr-Phe) models examining differences in compound-specific stable isotope ratios between genetic populations ECAG1 and ECAG2 and with day of year, measured in 60 killer whale skin samples collected between 2012 and 2022 from 8 locations across the northwest Atlantic. Exact sampling date was not known for five individuals, but they were known to be sampled in July and August (day of year 182-243). Variables are interpreted to be significant when the 95% Bayesian credible interval (L = lower limit, U = upper limit) did not overlap with zero. AAs that differed are in italics and indicated with an asterisk.

| **Model** | **Coefficients** | **Estimate** | **Est. Error** | **L-95% C.I.** | **U-95% C.I.** |  |
| --- | --- | --- | --- | --- | --- | --- |
| δ^13^C AA_ESS_ ~ genetic population + day of year | Ile Intercept | −13.1 | 0.93 | −14.92 | −11.27 |  |
|  | Leu Intercept | −23.83 | 1.05 | −25.91 | −21.76 |  |
|  | Met Intercept | −18.98 | 1.35 | −21.67 | −16.34 |  |
|  | Phe Intercept | −24.71 | 1.03 | −26.73 | −22.70 |  |
|  | Val Intercept | −17.11 | 3.02 | −23.03 | −11.15 |  |
|  | Ile Genetic Population ECAG2 | −0.38 | 0.23 | −0.84 | 0.08 |  |
|  | Ile day of year | −0.01 | 0.00 | −0.02 | 0.00 |  |
|  | *Leu Genetic Population ECAG2* | −*1.50* | *0.27* | −*2.03* | −*0.98* | * |
|  | Leu day of year | −0.01 | 0.00 | −0.01 | 0.00 |  |
|  | Met Genetic Population ECAG2 | *0.11* | *0.34* | −*0.55* | *0.78* |  |
|  | Met day of year | −0.01 | 0.01 | −0.02 | 0.00 |  |
|  | *Phe Genetic Population ECAG2* | *0.73* | *0.26* | *0.22* | *1.23* | * |
|  | Phe day of year | −0.01 | 0.00 | −0.02 | 0.00 |  |
|  | *Val Genetic Population ECAG2* | −*2.30* | *0.76* | −*3.80* | −*0.80* | * |
|  | Val day of year | −0.01 | 0.01 | −0.03 | 0.02 |  |
| δ^15^N_Glx-Phe_ ~ genetic population + day of year | Intercept | 11.59 | 2.11 | 7.44 | 15.75 |  |
|  | *Genetic Population ECAG2* | *2.51* | *0.54* | *1.45* | *3.56* | * |
|  | day of year | 0.00 | 0.01 | −0.02 | 0.01 |  |
| δ^15^N_Thr_ ~ genetic population + day of year | Intercept | −51.63 | 3.99 | −59.48 | −43.85 |  |
|  | *Genetic Population ECAG2* | *6.38* | *1.01* | *4.40* | *8.38* | * |
|  | day of year | 0.02 | 0.02 | −0.01 | 0.06 |  |
| δ^15^N_Thr-Phe_ ~ genetic population + day of year | Intercept | −65.15 | 3.73 | −72.49 | −57.81 |  |
|  | *Genetic Population ECAG2* | *7.50* | *0.95* | *5.63* | *9.35* | * |
|  | day of year | 0.02 | 0.02 | −0.01 | 0.06 |  |
